# Drugging Hijacked Kinase Pathways in Pediatric Oncology: Opportunities and Current Scenario

**DOI:** 10.3390/pharmaceutics15020664

**Published:** 2023-02-16

**Authors:** Marina Ferreira Candido, Mariana Medeiros, Luciana Chain Veronez, David Bastos, Karla Laissa Oliveira, Julia Alejandra Pezuk, Elvis Terci Valera, María Sol Brassesco

**Affiliations:** 1 Department of Cell Biology, Ribeirão Preto Medical School, University of São Paulo, Ribeirão Preto 14049-900, SP, Brazil; 2 Regional Blood Center, University of São Paulo, Ribeirão Preto 14049-900, SP, Brazil; 3 Department of Pediatrics, Ribeirão Preto Medical School, University of São Paulo, Ribeirão Preto 14049-900, SP, Brazil; 4 Department of Biology, Faculty of Philosophy, Sciences and Letters at Ribeirão Preto, University of São Paulo, Ribeirão Preto 14040-901, SP, Brazil; 5 Departament of Biotechnology and Innovation, Anhanguera University of São Paulo, UNIAN/SP, São Paulo 04119-001, SP, Brazil

**Keywords:** childhood cancer, kinases, chemical inhibitors, clinical trials

## Abstract

Childhood cancer is considered rare, corresponding to ~3% of all malignant neoplasms in the human population. The World Health Organization (WHO) reports a universal occurrence of more than 15 cases per 100,000 inhabitants around the globe, and despite improvements in diagnosis, treatment and supportive care, one child dies of cancer every 3 min. Consequently, more efficient, selective and affordable therapeutics are still needed in order to improve outcomes and avoid long-term sequelae. Alterations in kinases’ functionality is a trademark of cancer and the concept of exploiting them as drug targets has burgeoned in academia and in the pharmaceutical industry of the 21st century. Consequently, an increasing plethora of inhibitors has emerged. In the present study, the expression patterns of a selected group of kinases (including tyrosine receptors, members of the PI3K/AKT/mTOR and MAPK pathways, coordinators of cell cycle progression, and chromosome segregation) and their correlation with clinical outcomes in pediatric solid tumors were accessed through the R2: Genomics Analysis and Visualization Platform and by a thorough search of published literature. To further illustrate the importance of kinase dysregulation in the pathophysiology of pediatric cancer, we analyzed the vulnerability of different cancer cell lines against their inhibition through the Cancer Dependency Map portal, and performed a search for kinase-targeted compounds with approval and clinical applicability through the CanSAR knowledgebase. Finally, we provide a detailed literature review of a considerable set of small molecules that mitigate kinase activity under experimental testing and clinical trials for the treatment of pediatric tumors, while discuss critical challenges that must be overcome before translation into clinical options, including the absence of compounds designed specifically for childhood tumors which often show differential mutational burdens, intrinsic and acquired resistance, lack of selectivity and adverse effects on a growing organism.

## 1. Pediatric Cancer

The past two decades have witnessed tremendous advances in our understanding of cancer pathogenesis, with most neoplasms resulting from the accumulation of gains in function in proto-oncogenes and losses of tumor suppressors.

In the pediatric setting (between 0 and 19 years of age), cancer is defined as a group of several diseases that have in common the uncontrolled proliferation of abnormal cells that can occur in any region of the body. However, unlike adult tumors that are classified according to the primary site, the International Classification of Childhood Cancer (CICI) categorizes pediatric tumors into 12 main groups based on histological findings [1].

Of these, leukemias are the most frequent, representing 35% of all tumors that affect children and adolescents [2]. The second most common group is represented by lymphomas (20%), followed in descending order by tumors of the central (15%) and sympathetic (7%) nervous system, soft tissue sarcomas (6%), bone tumors (5%), kidney tumors (3%), germ cell tumors (3%), retinoblastoma (2%), carcinomas (2%), liver tumors (1%) and other rare pediatric neoplasms.

Several specific characteristics converge on the premise that childhood and adult cancer should be studied separately. First of all, most pediatric tumors have histological findings that resemble fetal tissues at different stages of development, being considered embryonic and carrying different levels of cell differentiation. Furthermore, the spectrum of tumors in the pediatric age group differs from that in adult patients. Medulloblastoma (MB), neuroblastoma (NB), rhabdomyosarcoma (RMS), Ewing’s sarcoma (EWS), osteosarcoma (OS), retinoblastoma (RB) and Wilms’ tumor (WT), which are the most frequent pediatric solid tumors, are rarely found in adulthood [3]

Moreover, latency periods are shorter in pediatric cancer, with several histologies presenting even shortly after birth. Such rapid proliferation in embryonic tissues relies mainly on genomic errors with lesser contribution of environmental factors [4]. Similarly, unlike what happens in adults, pediatric tumors are usually more aggressive with nonspecific signs and symptoms, confusing them with common childhood illnesses and making early diagnosis difficult.

Additionally, tumors in this age group show clear differences in their presentation, clinical course and response to treatment when compared to adult counterparts. Tumors of the EWS family, for example, in adults, in addition to presenting a more differentiated histology (PNET—primitive neuroectodermal tumor), manifest preferentially with greater volume at diagnosis, affecting soft tissues and with distant metastases, resulting in poorer survival. In children, EWS preferentially affects the bones of the extremities, have a smaller volume, and respond better to chemotherapy. Still, in this population, the chance of survival after 5 years (without metastasis at diagnosis) is 75%, significantly higher than that calculated for adults (50%) [5]. Similar observations have been reported for RMS. In a cohort of 1071 adults and 1529 children, for example, the survival rate in the first group was considerably lower (27% versus 61%), with tumors occurring in unfavorable locations and with rare histologies [6].

Another peculiarity of childhood cancer lies in the fact that specific histological subtypes and clinical behavior are also age-dependent, suggesting differential pathogenic mechanisms and underlying molecular alterations for tumor initiation and progression. The general incidence of acute lymphoblastic leukemia (ALL), for example, is highest in the 1–4-year-old age group, while the highest frequency of lymphomas occurs among adolescents (between 15 and 19 years old). Embryonic tumors (NB, WT, RB, etc.), on the other hand, share a descending incidence, which is highest early in life and almost dissipates after 5 years of age, while the incidence of bone sarcomas reaches a sharp peak at the time of the pubertal growth spurt [7].

Finally, with the methodologic refinements in the identification of genomic alterations, it has become increasingly evident that the spectra of mutations and the subsequent dysregulation of signaling pathways in pediatric neoplasms differ from those that occur predominantly in adult cancer. In fact, it has been stipulated that most pediatric tumors carry between 5 and 10 mutations; however, the average number of mutations in adult tumors varies between 33 and 66 (i.e., colon, breast or pancreas carcinomas) and increases up to 200 in tumors caused by mutagens (such as melanoma/ultraviolet radiation (UV) and lung cancer/smoking) and up to 1000 in tumors with defects in mismatch repair genes, as is the case of nonpolyposis colorectal cancer, among others [8]. In this regard, a recent integrative study based on whole-genome sequencing data from 24 tumor types (914 patients) showed that even though mutation frequencies (SNV and indels) vary between pediatric tumor types (from 0.02 to 0.49 per Mb), these were 14 times lower than in adult cancers [9]. Of note, a high prevalence of mutations affecting genes related to cancer predisposition syndrome are seen in children affected by different tumor types [10]. In addition, pediatric cancers are usually enriched by gene fusions, driving tumorigenesis and showing impact on both diagnostic and targeted-treatments [11].

In fact, this work identified 52 genes significantly mutated for childhood and juvenile tumors and 102 genes for adult tumors, 25 of which were shared by both groups. *TP53* was the most commonly mutated gene (4% of childhood tumors), followed by *KRAS*, *ATRX*, *NF1* and *RB1* (1–2% of tumors). In adult tumors, *TP53* was also the gene most affected by mutations, albeit tenfold more frequently. Furthermore, the burden of mutations increased with patient age except for tumors characterized by *kataegis* or *chromothripsis* events [9].

Moreover, the mutational identity may also vary. Glioblastoma (GBM) (grade IV astrocytoma), for example, is characterized by mutations in *PTEN* and epidermal growth factor receptor (*EGFR*) amplification in adults [12]; the pediatric counterpart more frequently presents mutations in the N-terminal tail of the histone variant 3.3 [13], in platelet-derived growth factor (*PDGF*) and its receptor (*PDGFR*) [14]. As previously described, gene fusions that are rare in adult tumors appear recurrently in pediatric tumors such as *BRAF/KIAA1549* in pilocytic astrocytoma (PA) [15], *C11orf95/RELA* [16] in supratentorial ependymoma (EPN), PAX3/FOXO1 and PAX7/FOXO1 in alveolar RMS [17], and variants involving the *EWS* gene (*EWS/FLI1*, *EWS/ERG* and others less frequent) in EWS [18]; the majority of these fusions involve transcription factors associated with the development/differentiation of the affected tissue.

More recently, with the establishment of cooperative study groups and progress in imaging associated with more accurate anatomopathological and molecular diagnosis, the mortality of children affected by cancer, especially those with leukemia and some types of solid tumors, has shown an important decline. Five-year overall survival rates increased from 56% in the 1970s to 77% in the following vicennial [19].

In accordance, today ALL represents the paradigm of curable cancer in children, with current overall survival rates exceeding 85% in most modern treatment protocols [20]. However, for some aggressive leukemia subtypes and certain solid tumor histologies, the persisting advances in biological characterization combined with new technologies in radiotherapy (RT), chemotherapy (CT) and supportive/rehabilitation care have resulted in marginal survival advantages, and cure rates have stagnated at around 70% [21]. Yet, in underdeveloped areas such as Eastern Europe, Africa and South America, these reductions in mortality have been less expressive, and a considerable portion of children with cancer fail to respond to traditional chemotherapy.

In this scenario, alternative rationally targeted pharmacological options are still needed to overcome clinical resistance, tumor progression, and prevent the adverse side effects of standard therapy.

## 2. Kinases as Cancer Drivers

The human genome encodes more than 500 protein kinases, enzymes responsible for turning protein functions “on” through the transference of γ-phosphate groups from ATP to one of their three amino acids with free hydroxyl groups: serine, threonine or tyrosine. Human protein kinases have been divided into nine classes which are further subdivided into families, and often subfamilies whose actions can alter up to 30% of all cell proteins [22]. The molecular shifts exerted by them through phosphorylation not only can affect the function of a given protein, but it can also stabilize it, localize it in a particular cellular compartment and modulate its association with other proteins [23].

Protein kinases may be triggered or deactivated in many ways, including cis- or autophosphorylation, binding with substrates or activator/inhibitor proteins. Once activated, they act as crucial regulators of many features of cell behavior and specialized functions by coupling reception of extracellular signals, intracellular signaling transduction and cellular responses [24].

**Figure 1:**
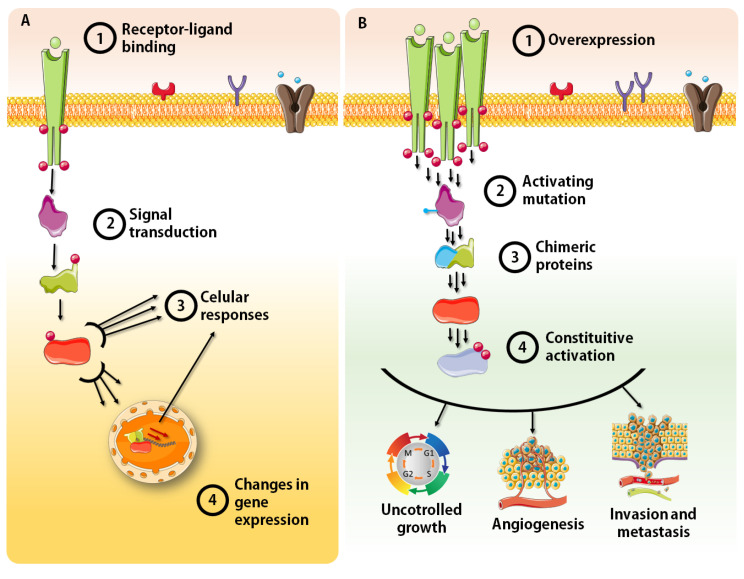
(**A**) Protein kinases may be triggered or deactivated in many ways, acting as key regulators of many features of cell behavior and specialized functions by coupling (1) reception of extracellular signals and (2) intracellular signaling transduction, leading to (3) direct cellular responses or (4) changes in gene expression. (**B**) In cancer, increased kinase activity may result from gene amplification (1,4), mutations that stabilize the kinase in an active conformation and destabilize cis-inhibitory interactions (2) and translocations that encode chimeric proteins with novel/increased activity (3). This figure was created using Servier Medical Art templates, which are licensed under a Creative Commons Attribution 3.0 Unported License; https://smart.servier.com (accessed on 14 December 2022).

Playing fundamental roles in cell division, survival and migration, their dysregulation is commonly associated with human malignancies and contributes to tumor initiation and all stages of cancer progression. In fact, innumerous mutations, translocations, and amplifications that result in constitutively overexpressed or active kinases have been demonstrated in many human cancers [23]. Mutations within the catalytic domain serve to stabilize the kinase in an active conformation and to destabilize cis-inhibitory interactions. Other domains can also be affected and elicit constitutive activity and hyperactive pathways as well. DNA translocations, on the other hand, predominantly create in frame gene fusions leading to chimeric proteins with novel/increased activity, leading to continued cancer cell growth and survival (Figure 1) [25,26]. Moreover, the dysregulation of different kinases has been repeatedly associated with tumor prognosis and categorized as a determinant of patient survival [27,28].

Hence, the so-called “Kinome” (the complete set of protein kinases encoded in the human genome—about 2% of all genes) has become an attractive target for the treatment of a variety of tumors. Even so, despite the great diversity, over the years, it has become more apparent that only certain kinases are among the most frequently occurring drivers of human cancer, including tyrosine receptors (RTK) (i.e., FGFR, EGFR, VEGFR, RET, MET, ALK), members of the PI3K/AKT/mTOR and Mitogen-Activated Protein Kinase (MAPK) pathways, along with central coordinators of cell cycle progression (i.e., cyclin-dependent kinases) and chromosome segregation such as polo-like and aurora kinases [25].

In this way, the present study aimed to present evidence of the involvement of these kinases’ dysregulation in the pathophysiology of pediatric tumors, their correlation with clinical outcomes and prospects of their inhibition through in silico analysis, along with an up-to-date revision of compound development and testing.

## 3. Protein Kinases in Pediatric Oncology and Their Association with Tumor Prognosis

**Figure 2:**
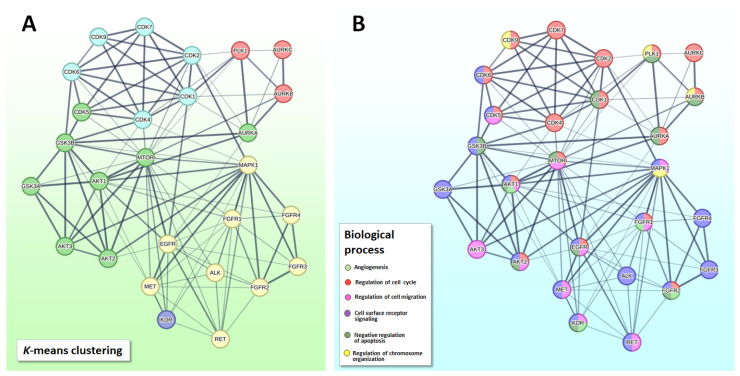
(**A**) Protein–protein interactions accessed through the software STRING v11.5 (available at https://string-db.org/ (accessed on 2 November 2022)). The parameters evaluated were text mining, experiments and databases. Network edges denote confidence and the minimum required interaction score was 0.700, considered high. (**A**) K-means clustering; (**B**) enrichment analysis for biological processes.

As stated above, substrate reversible phosphorylation by protein kinases is nature’s main molecular system for organizing cellular signal transduction and regulating cell metabolism, growth and differentiation. The phosphorylation state of a protein determines not only its function, subcellular distribution and stability, but also its interaction with other proteins or cellular components. Intrinsically, signaling pathways are remarkably complex and as our knowledge increases, it has become progressively evident that such molecular networks are not linear but contain modules of multi-protein complexes, many feedbacks, feedforwards and competing protein mechanisms that not only assemble at various intracellular compartments to process, integrate and transmit information that will ultimately specify a particular biological response, but also crosstalk with many other signaling pathways [29]. Thus, even though the kinases that were selected for this review will be treated separately, many, if not all, are directly or indirectly interconnected (Figure 2).

In this section, the different roles of protein kinases in oncogenic transformation and tumor prognosis in the pediatric setting were assessed by two different approaches: by a thorough search of published literature, and by a systematic search in publicly available data retrieved from expression arrays accessed through the R2: Genomics Analysis and Visualization Platform (http://r2.amc.nl (accessed on 15 October 2022)). For this, datasets were included if they met the following criteria: inclusion of pediatric samples (exclusively or in which adult variants could be omitted), normal counterparts and having information about clinical features of prognosis (Supplementary Figure S1). Datasets and probes are detailed in Supplementary Tables S1 and S2.

### 3.1. Published Evidence of Kinase Dysregulation in Pediatric Oncology

#### 3.1.1. Receptor Tyrosine Kinases (RTK)

Humans express 58 receptor tyrosine kinases (RTK) which function as entry points for many extracellular signals and the recruitment of the intracellular signaling networks that orchestrate a particular response [30].

These cell surface receptors possess multi-domain identical architectures that are made up of an extracellular ligand-binding domain (which differs between subfamilies), a single transmembrane helix and an intracellular region that contains a juxtamembrane regulatory region (composed of 40–80 amino acids), a tyrosine kinase domain (TKD) and a carboxyl (C-) terminal tail [31].

Generally, RTK activation occurs upon binding of ligands (i.e., growth factors or cytokines) to their extracellular domains. This interaction results in RTK non-covalent dimerization/oligomerization, which juxtaposes the cytoplasmic TKDs and facilitates autophosphorylation in trans of tyrosine residues in the juxtamembrane regulatory region, inducing conformational changes that serve to stabilize the active state of the kinase. Then, a second phase of tyrosine autophosphorylation occurs on phosphotyrosines that recruit downstream signaling proteins that typically contain Src homology 2 (SH2) or phosphotyrosine-binding (PTB) domains. The recruitment of these adapter molecules then initiates a cascade of RTK-specific pathways that determine cell fate [22,31,32].

About 20 different RTKs classes or subfamilies have been described [33]. Their activity is tightly regulated in normal cells; however, constitutive kinase activity acquired through mutation, overexpression and/or autocrine/paracrine stimulation has been strongly associated with pathological disorders, neoplastic transformation and metastasis [34]. Dysregulation of the epidermal growth factor receptor (EGFR/ErbB), the receptor for insulin (IR), the platelet-derived growth factor receptor (PDGFR), the fibroblast growth factor receptor (FGFR), the vascular endothelial growth factor receptor (VEGFR) and the hepatocyte growth factor receptor (HGFR/MET), for example, results in uncontrolled activation of multiple downflow signal transduction pathways and provides a strong drive toward malignancy [35]. Some of these oncogenes are paradigms of certain tumor types, as is the case of the amplification of EGFR/ErbB in breast cancer or MET overexpression in non-small cell lung cancer (NSCLC) [36,37]. Nevertheless, information about RTKs’ involvement in the pathophysiology of childhood cancer is less discernible, as illustrated below.

**FGFR.** The signaling cascades of fibroblast growth factor receptors (FGFR1, FGFR2, FGFR3 and FGFR4) play pivotal roles in the regulation of development and tissue repair and regeneration. These receptors are highly conserved and widely distributed and their dysregulation promotes tumor growth, survival and development of drug resistance, as well as the development of angiogenesis and immune evasion [38]. A recent pan-cancer next-generation sequencing profiling demonstrated that ~7% of cancers harbor gain-of-function FGFR aberrations, with varying frequencies between family members (FGFR1 > FGFR3 > FGFR2 > FGFR4) [39]. Gene amplifications or activating mutations have been observed in multiple cancer types, although they are most commonly detected in breast, lung, liver, stomach, uterus and bladder cancer [40]. In fact, most of the aberrations detected in the survey performed by Helsten et al. (2016) involved adult carcinomas, while the percentage of cases positive for *FGFR* aberrations in childhood tumors (NB and OS) was around 3% [39]. Additionally, *FGFR1* fusions (i.e., FGFR1-TACC1), TKD duplications and hotspot mutations (i.e., N5465K and K656E) are frequently observed in certain types of pediatric brain cancer, particularly dysembryoplastic neuroepithelial tumors (DNET) and PA [41]. Moreover, germline mutations in *FGFR1*, either complete or in mosaicism, may predispose low-grade central nervous system (CNS) tumors in children and adolescents [42,43].

Other research groups have also found correlations between altered expression of FGFRs and poor prognosis. *FGFR1* amplification, for example, was correlated with worse prognosis and poor response to chemotherapy in a large cohort of patients with OS [44]. Moreover, FGFR1 has been correlated with tumor development and lung metastasis in xenographic models, whereas its activation improved survival and radiation resistance, a phenotype which was reversed when FGFR1 was inhibited [45]. Similar to OS, *FGFR1* copy number gains are frequent in EWS, where patients with activating *FGFR1* mutations present higher incidence of metastatic disease [46]. In vitro, *FGFR1* suppression through interference RNA (RNAi) significantly reduced cell proliferation. Decreased xenograft tumor growth and 18F-fluorodeoxyglucose activity were also observed [47].

FGFR1 amplification and gene fusions have also been described in RMS with an active role cancer cell proliferation [48,49]. However, opposite expression patterns have been reported [50,51]. Nevertheless, it has already been shown that embryonal (ERMS) histologies present higher *FGFR1* expression levels compared to the alveolar forms (ARMS) [51].

Considering CNS tumors, upregulation of *FGFR1* has been associated with worse prognosis and shorter overall and recurrence-free survival in EPN and NB [52,53,54,55]. The analysis of FGFR1 protein abundance in human MB tissues, through an anonymized, validated MB, and cerebellum tissue microarray (TMA) and immunohistochemistry (IHC) found high levels of FGFR1 expression in 18% of the tumor tissues. In the case of gliomas, many mutations involving the *FGFR1* gene were described, one of which was associated with radioresistance [56,57,58,59,60]. Otherwise, in WT and MB, there is no published evidence.

Regarding FGFR2, information in the literature about its relevance in pediatric tumors is scarce. Besides correlations with higher tumor grade, radioresistance and poorer survival in gliomas [61,62,63], its phosphorylation (indicative of activation) was seen increased in NB samples (compared to normal tissues) and correlated with cisplatin resistance [62]. Alternatively, low expression of this kinase was observed MB [63] and in RMS when compared to normal myeloblasts [64]. Downregulation or undetectable expression of FGFR3 was also reported in RMS with no evidence of correlation with the clinical outcome [64]. However, a more recent study described a small population of FGFR3-positive cells as strongly tumorigenic with a stem cell-like phenotype [65].

*FGFR3* is also downregulated in WT [66]; however, in pediatric CNS tumors, opposite *FGFR3* expression profiles are observed. In NB, high expression levels are associated with worse overall survival and event-free survival (EFS) [67]. In glioma, its upregulation was associated with increased patient age [52], a feature that denotes a more invasive phenotype in adult counterparts [59]. Moreover, *FGFR3* amplification [68] and fusions (*FGFR3-TACC3*) seem to play a role in tumor metabolism and tumor growth promotion in low-grade gliomas (LGG) [69,70,71]. Such correlation with poor prognosis was also observed in EPN in which *FGFR3* was associated with shorter overall survival and shorter time to tumor recurrence [52].

Moderate-to-high expression of *FGFR3* mRNA was also observed in 80% of samples from EWS family tumors [71]. Mutations in this gene were also reported in circulating tumor cell samples [72]. However, neither of these studies presented information about correlations with clinical outcomes. Of note, when FGFR3 is downregulated in OS cell lines (by long noncoding, microRNA or iRNA), there is a reduction in tumor growth and angiogenesis, reinforcing its relevance to this disease [73,74,75,76].

Regarding *FGFR4*, little information about its prognostic value has been published in the pediatric setting, with a few reports on glioma, MB, RMS and NB. The prognostic value of this kinase in the first group was initially evaluated by in 2019 by Jimenez-Pascual and Siebzehnrubl, who did not find any correlations between FGFR4 expression and clinical outcomes [59]. Nevertheless, a recent evaluation of transcriptomic glioma datasets from The Cancer Genome Atlas (TCGA) revealed a direct association of high FGFR4 expression and dismal prognosis, progressively upregulated in recurrent tumors. In addition, the contribution of FGFR4 to the malignant phenotype of a highly aggressive GBM subgroup was further validated by increased viability, adhesion, migration and clonogenicity in vitro, along with abolished xenograft formation in mice and reduced invasiveness in zebrafish xenotransplantation models [76].

In MB, high *FGFR4* expression levels were observed in HD-MBO3 cells and in a small cohort (*n* = 12) of primary MB tissues, even though there was no validation upon TMA [63]. Of note, a pilot study based on an independent blinded set of 112 samples showed that protein levels of FGFR4 in urine, together with cadherin-1 (CADH1) and fibrinogen beta chain (FIBB), could be used to discriminate MB patients from healthy control patients with acceptable accuracy. Moreover, the authors reported a positive correlation of urine FGFR4 detection with the age of affected patients [77].

Additionally, FGFR4 overexpression in RMS contributes to the failure of cells to complete normal skeletal muscle development, leading to constitutive signaling and unregulated growth in correlation with poor differentiation [78,79,80,81]. *FGFR4* mutations in childhood RMS (7–8% of tumors) are more frequently observed within the kinase domain. From those, N535K and V550E increase autophosphorylation of the receptor and promote proliferation and metastatic potential when expressed in vitro [79]. High expression levels of FGFR4 have also been associated with advanced stage and poor survival in RMS [82,83]. Furthermore, FGFR4 has been reported as a downstream target of PAX3 and PAX3–FOXO1 [81] and thus is commonly altered in fusion-positive RMS [83] and a key contributor to RMS invasion and metastasis [84].

Last but not least, a germline polymorphism in the *FGFR4* gene (rs351855) which results in the expression of an arginine at codon 388 (Arg388), rather than the more common glycine (Gly388), is frequently associated with decreased survival rates, treatment resistance and more aggressive disease in a variety of malignancies, and is associated with an increased prevalence of NB in children [85], and this association may be linked to differences in FGFR4 degradation rates [86]. It was also observed that cases with the *FGFR4* AA genotype were 2.5 times more likely to have tumors with *MYCN* amplification compared with those with AG and GG genotypes, although such association was not statistically significant [85].

**EGFR.** The epidermal growth factor receptor (EGFR) (also recognized as HER-1 or ERBB-1) is a transmembrane glycoprotein of the ERBB receptor tyrosine kinase superfamily. Overexpression and/or enhanced activity of EGFR activate the downstream pro-oncogenic signaling, including the RAS-RAF-MEK-ERK and AKT-PI3K-mTOR pathways. These consequently activate several biologic expressions that proceed human cancer progression [87].

Overexpression of EGFR has been reproducibly detected in a large number of tumor samples and found to act as a strong prognostic indicator in head and neck, ovarian, cervical, bladder and esophageal cancers, correlating to poorer survival rates [88,89]. In the pediatric setting, however, there are few reports about its prognostic relevance. In gliomas, for example, fewer molecular alterations in the EGFR gene (mutations and amplifications) are observed in children when compared to adult counterparts [90,91,92,93,94,95]. *EGFR* gene amplification/overexpression is a genetic hallmark in adult GBM (observed ~40% of tumors) [95], whereas this feature is only observed in 25% of pediatric cases; nevertheless, it is associated in a similar manner with higher proliferation and increased tumor grade [96,97,98].

Likewise, high-level amplification and EGFR overexpression correlate with shorter event-free survival and relapse in high-grade EPN, being considered an independent prognostic marker for intracranial forms [99,100,101]. In MB patients, high expression of HER-2, another member of the EGFR gene family, was also associated with limited survival and metastasis [102,103]. Moreover, this RTK often co-expresses with HER-4 (more than 50% of samples), suggesting that HER-2/HER-4 heterodimerization may be of particular biological significance in this disease [101].

HER-2 expression was also reported to be associated with the aggressive behavior of NB and to significantly reduce survival [103]. However, a later study demonstrated that EGFR and HER-2 positivity are more frequently found in favorable histological risk groups, including younger age (≤18 months), localized disease, and favorable histological group [104]. Similar results were obtained by Izycka-Swieszewska et al. (2010), where HER-2-negative cases were more often found in the metastatic tumor group, associated with increased mitotic index and higher KI67 expression. *MYCN* non-amplified tumors were more often HER-2-positive than amplified tumors [105]. In contrast, higher expression levels of HER-4 are more often found in patients with metastatic disease [104].

For other pediatric tumors, the biological relevance of EGFR family members remains to be clarified. In EWS, for example, while HER-2 is not considered an important prognostic factor, an association of HER-4 and metastasis was found [106]. In OS, *EGFR* expression is common [107], but correlations between EGFR or HER-2 expression and clinical prognosis have been controversial, as no treatment improvements are achieved when EGFR is inhibited in pre-clinical and clinical trials [106].

In RMS, RB and WT, no strong associations of EGFR expression with clinical data have been found [108,109,110,111], although the ERBB family seems to be important for the malignant phenotype of RMS: ERBB1 sustains cell proliferation and growth, ERBB2 regulates myoblast cell transformation and survival and ERBB3 induces myogenic differentiation. Additionally, activation of ERBB2 coupled with inactivation of p53 induces RMS in animal models [106].

**VEGFR (KDR—kinase insert domain receptor).** The vascular endothelial growth factor receptor (VEGFR) family consists of three members: VEGFR1, VEGFR2 and VEGFR3 [112]. These receptors are established players in the formation of new blood vessels and the maintenance and remodeling of existing ones, during development and in adult tissues [113]. As such, in neoplastic growth, VEGFRs play an essential role in tumor neovascularization, providing oxygen and nutrition, and they facilitate tumor cells to metastasize and spread to distant organs [114]. Regarding pediatric cancer, VEGFRs have already been quantitatively evaluated in various types of refractory brain tumors [115]. Both VEGFR1 and VEGFR2 were detected in anaplastic astrocytoma tumor cells, MB and EPN samples [116,117,118,119,120]. Moreover, these receptors are frequently mutated and highly expressed in gliomas, NB and OS; in all cases, there is a negative correlation with unfavorable prognosis, advanced tumor stage, metastasis and shorter overall survival [121,122,123,124,125,126,127].

**RET.** Under normal conditions, the RET (“rearranged during transfection”) TKR pathway is activated by glial cell line-derived neurotrophic factor (GDNF) ligands that bind to coreceptors from the GDNF family receptor alphas (GFRαs), playing a major role during sympathetic and enteric nervous system development, where it signals toward proliferation, migration and differentiation. Apart from amplification, the constitutive activation of RET is caused by point mutations and gene rearrangements that drive malignancy in multiple tissues (i.e., papillary and medullary thyroid carcinomas and non-small cell lung carcinomas) [128,129,130,131].

RET rearrangements are also found in a high proportion of childhood papillary thyroid cancers [132,133,134]. Recently, it was observed that pediatric tumors (soft tissue sarcomas or medullary thyroid cancer) harboring either an RET-fused or RET-mutated pathogenic somatic alteration show clinical response to the RET inhibitor Selpercatinib [134].

RET mutations leading to dysfunctional ligand binding have also been described as the second most significant cancer-predisposing gene in the germline of patients with OS [135,136]. Moreover, RET is activated and can promote motility and colony formation in metastatic OS cells, contributing to the higher resistance of this tumor type to different chemotherapeutic agents [137,138,139,140]. Furthermore, NB cells and tumor samples demonstrated high RET expression levels [140], and its activation induces invasive spread NB in animal models [141].

**c-MET**. The mesenchymal–epithelial transition factor (c-MET), which is also known as hepatocyte growth factor receptor (HGFR), is an essential molecule for the survival and function of normal cells that promotes tissue remodeling and organ homeostasis [142]. MET’s gain of function either via overexpression, amplification, aberrant splicing or mutations is associated with the constant activation of downstream classic signaling pathways that sustain rapid proliferation, promote cell migration, angiogenesis and survival of cancer cells [143]. Moreover, recent evidence indicates that MET signaling participates in the acquirement of mesenchymal phenotype, tumor plasticity and adaptive responses to metabolic stress, contributing to the recurrence and metastatic dissemination of cancer cells [144,145].

The c-MET gene was first identified in the human OS cell line (HOS) that had been treated with N-methyl-N′-nitro-N-nitrosoguanidine (MNNG) as a gene able to transform normal fibroblasts [146]. Since then, its involvement in cancer establishment and progression has been repeatedly described in a variety of common and high-risk pediatric solid tumors, including not only sarcomas, but also gliomas, MB, NB, WT and hepatoblastomas, among others [147]. Of note, infantile hemispheric gliomas were recently recognized to be driven by different RTKs, including somatic fusions and alterations involving ALK, ROS1, NTRK and c-MET [148]. Especially in anaplastic, diffuse and PA, c-MET levels often correlate with tumor grade [149].

Cytoplasmic c-MET immunoreactivity is also associated with poor clinical outcome, and tissues with overexpression often exhibit higher vascular proliferation and proliferative index [150,151,152]. Similar phenotypes have been observed in NB, where overexpression of this receptor promotes invasion and is associated with advanced metastatic stage [153,154].

Likewise, high levels of MET protein are associated with increased proliferative activity invasion and metastasis in WT [155,156], and represent a risk factor for invasion in RB [156].

In childhood sarcomas, several studies have pointed out c-MET as a promising biomarker capable of predicting poor prognosis. Forced expression of MET in primary osteoblasts induces transformation and is essential for the maintenance of the cancer phenotype [157], while loss-of-function approaches in OS cell lines (143B and U2OS) demonstrated that this oncogene promotes cell proliferation, migration and invasion, and inhibits cell apoptosis [158]. However, the study of genomic status of MET and other genes implied in ossification processes in a cohort of 91 children and teenagers showed that *MET* is mainly deleted, although the clinical subgroup with MET amplification presents worse outcomes [159].

In EWS, modest to high MET cytoplasmic/membranous expression is detected in the majority of tumor samples and is significantly correlated with a poor overall survival. However, there were no significant correlations between MET expression and clinical characteristics, including tumor stage, tumor location and age at diagnosis. The same group also detected genetic alterations that result in the formation of truncated MET proteins in 5% of patients and in two cell lines (ES-2 and ES-7) [160].

Finally, this RTK is overexpressed in RMS tumor samples [161,162,163] and cell lines, contributing to the metastatic and invasive features of this tumor type [163,164].

**ALK.** This RTK was first described in 1994, as a fusion partner in the t(2;5)(p23;q35) chromosomal translocation characteristic of anaplastic lymphoma from which takes its name [165]. In general, ALK activates multiple signaling cascades, such as the PI3K-AKT, CRKL-C3G, MEKK2/3-MEK5-ERK5, JAK-STAT and MAPK pathways, and its role in cancer may vary due to many factors, including not only its fusion partners (more than 30 described so far), but also the tumor type or its genetic background (its effects on NB, for example, are dependent on *MYCN* status) [166].

Next-generation sequencing has revealed the presence of several *ALK* mutations in pediatric cases with RMS, EWS, WT and OS [161,167,168,169,170]. Most mutations are located within the kinase domain and can be divided into three groups: ligand-independent mutations (F1174I, F1174S, F1174L and R1275Q), ligand-dependent mutations (D1091N, T1151M and A1234T) and a kinase-dead mutation (I1250T) [167].

Germline gain-of-function point mutations are observed in half of hereditary NB and in 9% of the sporadic forms [170], and correlate with high risk and poor prognosis [171,172], mainly because both the wildtype and mutant forms of ALK induce MYCN transcription and potentiate its oncogenic activity in this tumor type [173]. Other ALK-driven pediatric tumors include infantile hemispheric gliomas [149,174], inflammatory myofibroblastic tumors, renal cell carcinomas [167] and pediatric mesotheliomas [175].

ALK in-frame translocations have been described in EPN and EWS as detected by fluorescent in situ hybridization with the brea-apart of 5′ and 3′ probes [160,176]. Finally, ALK expression is strongly associated with the WNT-activated MB subtype in which, differently from other pediatric tumors, it represents an independent indicator of good prognosis for medulloblastoma patients [177].

#### 3.1.2. PI3K/AKT/mTOR Pathway

The phosphatidylinositol 3-kinase (PI3K)/Akt/mammalian target of rapamycin (mTOR) signaling pathway is among the best investigated in human biology, and is considered a key player in both physiological and pathological conditions [178].

The first step of activation of this pathway consists of the recognition of various growth factors and cytokines by RTKs localized at the cytoplasmic membrane. Then, these receptors dimerize and undergo autophosphorylation, activating GRB2 (Growth Factor Receptor Bound Protein 2) and SOS (Ras/Rac Guanine Nucleotide Exchange Factor). These activate Ras through the exchange of GDP with GTP, which then phosphorylates and activates the PI3K [179]. Active PI3K catalyzes the conversion of PIP2 (phosphatidylinositol 4,5-bisphosphate) to phosphatidylinositol-3,4,5-trisphosphate (PIP3), a second messenger that binds and recruits AKT to the cell membrane, which causes a conformational change in AKT and makes it more accessible to the PDK1-mediated phosphorylation of Thr308, followed by the phosphorylation at serine-473 by the mTOR2 complex [180]. This activation then induces a detachment of AKT from the inner surface of the plasma membrane, and the relocation to the nucleus where AKT isoforms phosphorylate and modulate the activity of several transcription factors. More than 100 different effectors have been described, including cyclin-dependent kinase inhibitor kip1 (p27kip1) through the FOXO family of Forkhead transcription factors, glycogen synthase kinase 3 (GSK3) and cell cycle stimulators, including cyclin D1 and c-Myc. AKT can regulate apoptosis by the inhibition of Fas ligand (FasL), BCL2-associated death promoter (BAD), BCL-2-interacting mediator of cell death (BIM) or BCL-2-associated X-protein (BAX), and by the degradation of p53 [181].

AKT also activates mTOR1, which has many different targets, including translation transcription factors that initiate transcription of genes associated with cell survival and growth and factors associated with hypoxia and angiogenesis (Figure 2) [182].

In cancer, this pathway can be dysregulated as a result of the activation of upstream oncoproteins including RTKs, by the loss or decreased level of its negative regulators such as the phosphatase PTEN (phosphate and tensin homolog deleted on chromosome 10), or directly through mutation and overexpression [183].

Undeniably, this pathway is activated in a wide variety of tumors (i.e., prostate, breast, lung and leukemia, among many others), leading to a profound disturbance of cell growth control, metabolic reprogramming and invasion/metastasis, as well as the suppression of autophagy and senescence [179,184,185,186,187,188,189]. Moreover, increasing evidence points to its critical participation in the maintenance of stemness in a variety of cancers, contributing directly to recurrence and chemoresistance [189].

In the pediatric setting, the PI3K/AKT/mTOR signaling axis has been described as abnormally activated in both hematologic and solid tumors, mainly as a consequence of chromosomal gains amplifying the AKT1 gene (described in rare cases of leukemia) [190,191,192] or the aberrant expression of PI3K isoforms [193]. Below, the involvement of this pathway’s individual members in childhood tumors is explored.

**PI3K.** PI3K is a group of plasma membrane-associated lipid kinases, consisting of three subunits: p85 regulatory subunit, p55 regulatory subunit and p110 catalytic subunit. According to their structure and substrate specificity, these kinases are grouped into three categories (classes I, II and III) [194,195]. PIK3CA (phosphatidylinositol 3-kinase, catalytic, α-polypeptide), the gene encoding the p110α subunit, is frequently mutated in ~30% of common human cancers and has been studied most thoroughly [196,197]. Although numerous mutations in this gene have been described, most gain-of-function mutations cluster around two hotspots at exons 9 and 20 [197]; however, contrasting roles for mutations at each exon have been described depending on the tumor type [198,199]. PI3K amplifications have also been frequently described and correlated with aggressive phenotypes, chemoresistance and poor prognosis [200,201,202,203].

The prognostic power of PI3K alterations in childhood cancer has been less explored. In MB, however, although no mutations have been detected [196], the p110α isoform is typically overexpressed, promoting cell proliferation, chemoresistance and migration [204,205,206].

Dysregulation of PI3K signaling is also considered an important player in gliomagenesis, with key roles in regulating cell movement and thus contributing to the highly invasive phenotype of GBM. Compared with normal human astrocytes, overexpression of PI3K p110 catalytic subunits, p85 regulatory subunits and phosphorylated Akt (Ser473) was also detected in two pediatric GBM cell lines (GBM6840 and GBM2603) [206]. Likewise, overexpression of the catalytic p110δ and regulatory p85α isoforms was also detected in a panel of primary NB samples and cell lines with active roles in cell growth and survival. Especially, p110δ was correlated with *MYCN* amplification [207]. However, this gene is significantly lower in NB samples with loss of heterozygosity at 1p36 and associated with poor clinical outcome [208,209,210].

The regulatory domain of PI3K, p55, is upregulated in sarcoma stem-like cells and promotes invasion, migration and chemotherapy resistance [210]. In EWS, despite variable expression levels between samples, this positive regulator has an oncogenic role [211]. Moreover, p55 analysis on a human sarcoma TMA (that includes two EWS samples) performed by Yoon et al. demonstrated a 4.1-fold increase compared with normal tissues [210].

**AKT (PKB).** AKT or PKB (protein kinase B) is a serine/threonine kinase that functions as an important regulator of cell growth, survival and glucose metabolism. There are three isoforms of mammalian AKT which are encoded by different genes [212]. AKT1 and AKT2 are ubiquitously expressed and are mostly involved in regulating cellular survival and protein synthesis, involved in glucose transport through the insulin signaling pathway, respectively. The function of AKT3 is not yet fully understood and its expression is almost entirely limited to the nervous system tissue [213,214,215]. Nevertheless, it has been reported that despite the high similarity, AKT isoforms exert non-redundant specific effects under physiological and pathological conditions [215].

Gain-of-function mutations in all three AKT genes have been identified in ~40% of breast, colon, melanoma and ovarian cancers [216,217]. G49A mutations affecting the pleckstrin homology domain of AKT1, for instance, were identified in ~5% of breast, colorectal and ovarian cancers [217]; however, this mutation was not detected in any of the 100 cases of GBM or 75 cases of MB analyzed by Schüller et al. in 2008 [218]. In the pediatric population, MB samples show p-AKT, and cell lines have shown to be crucially dependent on PI3K/AKT pathway activation; however, the phenotype was attributed to PTEN inactivation as a result of the loss of heterozygosity of chromosome 10q or promoter [219].

In pediatric sarcomas, Akt1 has been shown to contribute to the maintenance of the undifferentiated state of myoblasts pointing towards Akt signaling as a critical RMS nodal point [220]. The AKT pathway is also considered to be an important mediating survival signal in EWS [221], Likewise, an increasing body of evidence has shown that this pathway is frequently hyperactivated in OS and contributes to disease initiation and development, including tumorigenesis, proliferation, invasion, cell cycle progression, inhibition of apoptosis, angiogenesis, metastasis and chemoresistance [222,223,224]. The AKT2 gene is significantly upregulated in chemoresistant OS cell lines [224] and tumor samples, being significantly associated with positive recurrence, the presence of metastasis, poor response to chemotherapy and shorter EFS and overall survival [224]. Moreover, the AKT3 isoform was evidently upregulated in OS tissues and positively associated with tumor size [225].

AKT2 also plays an important role in NB by regulating N-myc expression. Of note, attenuation of this AKT isoform impaired proliferation and anchorage-independent cell growth, and decreased the secretion of angiogenic factor VEGF and decreased the potential to metastasize to the liver in vivo, thus implicating AKT2 in multiple aspects of NB initiation and progression [226].

**mTOR.** mTOR is a serine/threonine protein kinase that forms the catalytic subunit of two structurally and functionally distinct protein complexes, known as mTOR Complex 1 (mTORC1) and 2 (mTORC2) [227]. mTORC1 consists of mTOR, Raptor, GβL (mammalian lethal with SEC13 protein 8) and domain-containing mTOR-interacting protein (DEPTOR), and plays active roles in integrating various signals that specify the availability of growth factors, nutrients and energy in order to endorse ribosomal biogenesis, protein translation during cell growth and the expression of metabolism-related genes, while inhibiting apoptosis and autophagy [228]. mTORC2, on the other hand, is composed of mTOR, Rictor, GβL, Sin1, PRR5/Protor-1 and DEPTOR, and regulates cytoskeletal dynamics, ion transport and promotes cell proliferation and survival through the activation of Akt [229,230,231].

mTOR is frequently improperly activated in human cancers and results in alteration of both mTORC1 and mTORC2 signaling pathways, leading to increased cell proliferation and decreased apoptosis. However, among 33 mTOR activating mutations identified in 2014 by Grabiner et al. [231], those that were functionally tested in vitro conferred varying degrees of pathway activation, and, most importantly, a few displayed some substrate preference towards the eukaryotic translation initiation factor 4E binding protein 1 (4EBP1) and ribosomal protein S6 kinase (S6K1), or towards AKT1, implying that such mutations had distinct effects on mTORC1 or mTORC2. Specifically, 4EBP1 activation by mTOR1 is a major contributor to accelerated cell proliferation or increased cell survival; the so-called eIF4E-sensitive mRNAs code for various cell cycle and apoptosis regulators, including cyclins D1 and D3, CDK2, MYC, PIM1, Bcl-2, Bcl-xL and VEGF, among others [232,233,234].

mTOR overactivation is observed in many childhood tumors, including EPN, MB and PA, high-risk NB, WT and RB, leading to worse prognosis and survival [105,235,236,237,238,239,240,241].

Constitutive activation of the mTOR pathway, predominantly through mTORC2, is observed in EWS, with active roles in metastasis formation [241,242,243,244]. The metastatic behavior of OS is also dependent on the PI3K/Akt/mTOR cascade, in which mTOR contributes to cellular transformation and poor cancer prognosis via its downstream effectors S6K1, 4EBP1 and eIF4E [245,246]. In RMS, lower disease-free or overall survival is also associated with the activation (phosphorylation) of multiple interconnected Akt/mTOR pathways [246]. Of note, rapamycin treatment can greatly reduce the growth of cell lines derived from these three sarcoma types [242].

**GSK-3.** The glycogen synthase kinase is a ubiquitously expressed serine/threonine kinase existing as GSK-3α and GSK-3β isoforms (encoded by separate genes), both of which are downstream effectors of AKT [247]. Differentially from other kinases, GSK3 is one of the few whose activity tends to be high in resting cells, and exposure of cells to growth factors, serum or insulin results in its catalytic inactivation [248].

The GSK3 kinases are pleiotropic, phosphorylate many proteins, and interact with multiple signaling pathways [249]. These kinases can modify the activity of transcription factors that have profound regulatory roles in cellular proliferation (such as p53 and NF-κB), transcription factors important for epithelial–mesenchymal transition (EMT) (i.e., Snail) and pro-apoptotic molecules including BCL2 and BAX [250]. Therefore, aberrant activity of GSK3s can result in many diseases and disorders and influence oncogenesis and metastasis [251]. However, since GSK3s are involved in a wide range of signal transduction cascades and a plethora of cellular functions [252,253], their roles in cancer establishment and maintenance can deviate from their chief tumor suppressor effects and also promote neoplastic transformation [254,255,256,257]. This dichotomy is also observed in the pediatric setting. Strong evidence provided by Wang et al. (2008) [257], for example, demonstrated that GSK-3 activity is essential for the maintenance of MLL-positive leukemias. MLL rearrangements are in >70% of infant leukemia, and irrespective of the translocation partner, they are associated with poor clinical outcomes [258,259,260]. Alternatively, as a key suppressor of the Wnt, Hedgehog and Notch pathways GSK3 has attracted much scrutiny. Within these pathways, this kinase is critical in regulating the turnover of the effectors β-catenin, c-Myc and c-Jun, targeting them for degradation/inactivation, and this inhibits proliferation and stem cell maintenance [250].

The literature about the prognostic value of GSK3A in pediatric cancer is scarce. No evidence was found in the literature about its involvement in EWS, OS, RMS, WT, RB, NB and EPN. However, its role in MB has been explored in vitro and in vivo, showing to be important for cell proliferation and tumor growth [260], a phenotype that seems to be similar in pediatric glioma [261].

On the other hand, the role of GSK3B is more extensively studied. In EWS, this gene can either promote or impair tumor growth and is associated with good prognosis [262,263,264,265]. Interestingly, in OS, the same gene acts as an oncogene [265], and is associated with worse response to neoadjuvant chemotherapy [266].

The oncogene status also occurs in alveolar RMS, where GSK3B is directly involved in regulating the transcriptional activity of PAX3/FKHR [267] at the same time that the chimeric protein enhances GSK3B activity, which in turn represses MYOGENIN, a member of the muscle regulatory factor family that orchestrates the terminal differentiation step of skeletal muscle cells [268]. GSK3B is also involved in the maintenance of undifferentiated phenotypes in ERMS [269].

GSK-3B is highly expressed in high-risk NB; however, its expression is not associated with clinical stage, survival or other clinicopathological parameters [270]. GSK-3B has also been involved in the protection of NB cells against chemotherapy by regulating NF-kB signaling [271]. In this regard, several authors have shown that GSK3 inhibitors are able to regulate MYCN mRNA levels and reduce NB cell viability through multiple mechanisms, including p53 and Wnt signaling, BDNF/TrkB/PI3K/Akt, suggesting that targeting this kinase might potentiate chemotherapy [271,272,273,274].

Alternatively, a predominantly tumor-suppressive role for GSK3B is observed in MB, in which its accumulation leads to the downregulation of GLI, the most important activator and driver of the SHH medulloblastoma subtype [274]. Constitutive phosphorylation leading to GSK3β activation improves cell survival and contributes to malignant transformation [275]. Dysregulated GSK3B also sustains the survival, immortalization, migration, invasion and maintenance of stem cells in glioma [276,277,278].

#### 3.1.3. MAPK Pathway

The mitogen-activated protein kinases (MAPKs) comprise a group of serine–threonine protein kinases that control numerous cellular processes, including proliferation, differentiation, apoptosis, survival, inflammation and innate immunity [278]. In mammals, MAPKs include three main signaling axes, namely c-Jun NH2-terminal kinase (JNK), p38 MAPK and extracellular signal-regulated kinase (ERK), each of which exists in several isoforms [279].

This pathway mediates intracellular signaling triggered by extracellular stimuli such as growth factors and cytokines (ERK), or by intracellular stimuli such as genotoxic, osmotic, hypoxic, oxidative or endoplasmic reticulum (ER) stress (JNK and p38), for example [280,281].

The general cascade pattern includes initial activation of MAP4Ks (membrane-bond GTPases such as RAS, RHO, RAN, RAB and ARF) by RTKs, which phosphorylate intermediate MAP3Ks (i.e., RAF, MEKK). These then mediate phosphorylation and activation of MAP2Ks (MEK1/2—mitogen-activated protein/extracellular signal-regulated kinases, MKK4/7), followed by the positive phosphorylation of MAPKs (ERK1/2, p38 or JNK). Once activated, MAPKs phosphorylate diverse substrates, including transcription factors such as c-Jun, c-Myc, P53 and ATF2, thereby giving rise to the various cellular responses [282,283]. p38 MAPKs have also emerged as important modulators of gene expression by regulating chromatin modifiers and remodelers [281].

Thus, compromised MAPK signaling contributes to the pathology of a wide spectrum of human malignancies [282]. However, while the roles of JNK and p38 pathways are elusive [284,285,286,287,288,289,290], dysregulation of the RAS/RAF/MAPK(MEK)/ERK pathway explicitly drives the oncogenic process [291].

In fact, many of the cancer-associated mutations of components of MAPK signaling pathways have been found in RAS. Missense gain-of-function mutations in all three RAS genes (HRAS, KRAS and NRAS) are found in ~30% of all human cancers [292,293,294]. Other perturbations in GDP–GTP regulation, persistent receptor tyrosine kinase-mediated activation of GEFs, and miRNA deregulation are additional mechanisms of RAS activation in cancer and result in constant input signals with downstream kinases [291,295].

In this regard, the frequency of genomic alterations in the MAPK pathway as a whole parallels the direction of the signaling cascade: RAS > BRAF > MEK, and ERK mutations are exceptionally rare [296,297].

**RAF.** RAF has three isoforms (ARAF, BRAF and CRAF/RAF1), sharing a high similarity of domain organization. These cytoplasmic serine/threonine-specific protein kinases are essential effectors of the MAPK pathway through the association with activated RAS. This binding leads to their homo- or heterodimerization and activation with the phosphorylation of ERK.

Altered activation of RAF members results in increased proliferation in a broad range of human tumors [298,299]. The most common gain-of-function mutation in the members of the family occurs in BRAF codon 600, in which a valine is substituted for glutamic acid (BRAF-V600E). This point mutation is notably widespread in pilocytic astrocytoma (15%), melanomas (63%) and papillary thyroid carcinomas (more than 50%) [300,301]. Overexpression of full-length RAF or the truncated catalytic domain also leads to hyperactivated ERK signaling, resulting in increased malignant behavior [302].

**ERK1/2**. ERK1 and ERK2 are the prototypes of the eight isoforms of ERK and are activated by MAPK/ERK kinase (MEK) 1 or 2. Upon activation, ERK detaches from cytoplasmic anchoring proteins and translocates to the nucleus to exert its transcriptional regulation. Despite the well-recognized importance of ERK activation in cancer malignancy, mutations in these genes have rarely been reported as drivers in human cancers. Nonetheless, The Human Protein Atlas classifies them with enhanced expression compared to normal tissues.

The most compelling evidence of MAPK activity in cellular processes contributing to the development and progression of childhood tumors is represented by the duplication/rearrangement of *BRAF* at 7q34 leading to *KIAA1549:BRAF* fusion product, which is the most common molecular alteration in sporadic PA, occurring at the highest frequency in tumors of the posterior fossa [303,304,305]. ERK2 was identified as differentially expressed in tumor samples compared to normal tissues [306]. RAS/MAPK activation was associated with metastatic disease in MB [307,308]. Associations of ERK hyperexpression with distant metastasis and poor overall survival were also reported for childhood sarcomas, including RMS and OS [309,310,311,312]. For other pediatric tumors, activation of this pathway results from their interaction with dysregulated microRNAs [313,314].

#### 3.1.4. Cell Cycle Kinases

The cell cycle is a complex and well-ordered series of irreversible events through which a cell duplicates its DNA and grows to produce two daughter cells with identical genomes [315,316]. Transitions from one state to the next are driven by many oscillating regulators that determine whether cells proceed through G1 into the S phase, and from G2 to M, each of which are characterized by distinct molecular features and functional outputs [317]. Central to this process are the cyclin-dependent kinases and other key regulators such as kinases from the Polo and Aurora families.

##### Cyclin-Dependent Kinases

Cyclin-dependent kinases (CDKs) comprise 13 key regulatory enzymes involved in cell proliferation through the regulation of cell cycle checkpoints and transcriptional events in response to extracellular and intracellular signals. These intracellular serine/threonine kinases, whose catalytic activities are regulated by interactions with the adaptor molecules cyclins and CDK inhibitors (CKIs), orchestrate the evolution through the sequential phases, including entry into the cell cycle from quiescence, the G1/S phase transition, DNA replication in the S phase, nuclear breakdown, chromosome condensation and segregation, and cytokinesis [318].

CDKs coordinate cell cycle regulation at different stages to ensure the coherence, integrity and maintenance of every step in a sequential manner. CDK1 and CDK2, for instance, are necessary to direct the transition from S to G2, but only CDK1 governs the G2/M transition and mitotic progression [319]. Other CDKs regulate the cell cycle indirectly by activating other members of the family (CDK7, CDK20) or transcription (CDK7, CDK8, CDK9, CDK12, CDK19) [320,321].

Changes in the expression and regulation of CDKs induce unscheduled proliferation and chromosomal instability, well-known hallmarks of cancer and tumor aggressiveness [322,323,324,325,326,327]. Amplification or mutation of genes encoding CDKs, cyclins or endogenous inhibitors of CDKs have been described in many solid cancer types, and are recurrent events in the development of breast cancer [328] and GBM [329], for example. Such alterations are also described as molecular drivers in childhood tumors [330,331,332,333,334].

**CDK1**. Cyclin-dependent kinase 1 (CDK1) is vital in governing cell division and transition from G2 to the M phase [335]. Its dysregulation is common in many tumors of diverse origins, leading to chromosomal instability via replication stress and enhanced proliferation of cells. A recent pan-cancer integrative analysis based on TCGA and GTEx databases performed by Liu et al. (2022) showed that CDK1 expression levels are increased in many tumor types when compared to normal tissues and are generally associated with poor clinical prognosis [336]. For example, CDK1 expression is positively and highly associated with advanced cancer stages in lung and endometrial cancer [337,338]. Similar results were reported for other tumors, such as breast [339,340,341]. Moreover, CDK1 expression is positively correlated with the expression of the stemness marker SOX2, indicating a direct action on tumor maintenance and chemoresistance [342,343].

Regarding pediatric tumors, this kinase is associated with lower overall survival and EFS rates in EPN [344,345], RMS [346,347] and NB [348]. In silico analyses have also demonstrated that CDK1 is differentially expressed in RB [349] and plays a key role in the development of OS, since its negative regulation or depletion leads to significant decreases in proliferation while inducing apoptosis [350,351,352,353].

Furthermore, according to the literature, a well-described relationship exists between CDK1 and EWS, WT and high-grade gliomas (HGG). Specifically, this kinase expression has been directly associated with tumor progression [354], being considered a hub gene for GBM [355].

**CDK2.** Cyclin-dependent kinase 2 (CDK2) drives the entry of cells into the S and M phases of the cell cycle. Except for a few exceptions (i.e., testis), the majority of normal tissues have low expression of this serine/threonine kinase [356], and its activity is not essential for normal development [357]. However, CDK2 has been associated with cancer progression and aggressiveness across several malignancies [339,358], contributing not only with genomic instability and under-replication of DNA in the late S phase [359] but also through interactions with other proteins in a wide range of biological processes such as DNA damage response, intracellular transport, protein degradation and signal transduction, among others. Differentially from other kinases, several investigations have demonstrated that CDK2 is not upregulated or amplified; instead, its dysregulation results from altered binding partners or alterations due to post-translational modifications [360]. In tumors with MYCN overexpression, as is the case of NB, interaction with CDK2 appears to be critical for senescence avoidance and immortalization [361], being associated with worse prognosis and considered a suitable therapeutic target in this tumor type [362,363]. CDK2 inhibitors effectively induced cell cycle arrest or apoptosis in MYC-driven MB [364].

The literature also shows overexpression of CDK2 in HGG compared to normal tissue and low-grade forms, with a direct association with worse prognosis due to immune cell infiltration [365]. Moreover, this kinase plays a central role in the development of NB. Even though there is no well-established relationship between this kinase and the development of this tumor type, Zhang et al. (2016) [366] demonstrated that the inactivation of TAZ (a biomarker of aggressiveness in RB through miR-125a-5p) inhibited proliferation and tumor formation by decreasing cyclin E and CDK2 expression [367]. Similarly, although there is no clear and explicit description in the literature about the relationship of this kinase with RMS, Knudsen et al. also discussed the relationship between sustained CDK2 levels in RD cells irrespective of the exposure of cells to differentiating culture media, explaining the inability of those cells to arrest growth and thus contributing to oncogenesis [368]. Moreover, there are reports of apoptosis induction in several sarcoma cells after CDK1 and CDK2 co-depletion [369,370]. Of note, in a microarray-based study, CDK2 was found to be overexpressed and associated with poor prognosis in EWS [371].

**CDK4/6**. Cyclin-dependent kinases 4 and 6 are highly homologous key components of the cell cycle to drive the passage from G1 to S phase. Upon interaction with any D-type cyclin (CCND1, CCND2 or CCND3), these interphase kinases phosphorylate Rb to release E2F from Rb and initiate the transcription of genes required for cell cycle progression. Besides proliferation, other roles of cyclin-D/CDK4/6 have been confirmed, including the regulation of senescence, apoptosis, migration/invasion and angiogenesis [372].

Consequently, the complex CCND/CDK4/6 shortens G1, and hence, its constitutive activation represents a driving force of tumorigenesis. These proteins are generally concurrently studied and, in many cases, they present themselves with similar patterns, being simultaneously dysregulated [373,374].

CDK4 was identified as a major risk factor for disease progression in Paget’s disease [375] and its overexpression and/or hyperactivation is implicated in many types of human cancers [376,377,378,379,380,381]. Point mutations at the CDK4 locus (CDK4R24C) have also been reported [382].

Co-overexpression of both CCND1 and CDK4 is common in hepatoblastoma, a rare malignant liver tumor of childhood, and usually positively correlated with tumor recurrence [383]. In parallel, enhanced kinase activity of CDK6 has been associated with other childhood tumors [384]. This kinase plays an important role during hematopoiesis and is frequently altered in hematological malignancies of different immunophenotypes [385,386]. MLL-AF9 oncofusions in myeloid leukemia, for example, induce high CDK6 levels, acting as a blocker of myeloid differentiation and contributing to the maintenance of an immature phenotype [387]. This MLL fusion-driven activation of CDK6 (through MLL-AF4 and MLL-ENL) has also been described in infant leukemia [388].

Likewise, CDK4 and CDK6 have been described with similar frequencies in WT compared to normal mature kidneys, even though only CDK4 showed correlation with relapse [389]. However, a more recent study by Haruta et al. (2019) showed that WT samples with chromosome 12 trisomy does indeed show upregulation of this kinase, but that stronger expression is associated with better overall survival [390].

Activation of the CDK4/6 pathway is also a powerful driver of sarcomagenesis [391]. Amplification of 12q13-15 also occurs in OS, and a recent copy number analysis of pediatric high-grade OS detected a recurrent gain of chromosome 12q14.1 in ~25% of samples, which resulted in CDK4 overexpression. In vitro, higher expression of CDK4 was considered a predictive biomarker for resistance to cisplatin [392]. Indeed, elevated CDK4 expression is correlated with metastasis potential and poor prognosis in this tumor type [393,394,395]. Consistent with these findings, a recent study demonstrated that about 50% of OS samples present CDK4 somatic variants, 9.5% of which were identified as gain-of-function CNVs correlated with metastasis and death [396].

The inhibition of CDK4/6 also represents a promising precision medicine-guided therapy for other childhood sarcomas. A parcel of PAX3/PAX7-FOXO1-positive RMS tumors with amplification of the chromosomal region 12q13-q14, for example, also presents elevated CDK4 levels relative to non-amplified, fusion-negative forms [397,398]. In addition, in Brazilian cohorts, amplification or overrepresentation of CDK4 was evinced through qRT-PCR and immunoreactivity in both forms of RMS (ERMS and ARMS), along with several leiomyosarcoma samples [399,400]. Similarly, using a human TMA, Saab et al. (2007) demonstrated CDK4 expression in 82% of ARMS and 63% of ERMS tumors [401]. CDK6 was detected at high levels in six RMS-derived cell lines, reinforcing the prospects of its inhibition as a therapeutic opportunity [402]. In a similar manner, a shRNA-based screening demonstrated that CDK4 (together with CCND1) is required for survival and anchorage-independent growth in EWS [403].

More recently, a systematic evaluation of CDK4/6 as targets in a series 16 pediatric cancer types indicated that further preclinical evaluations are still needed to affirm the dependence of tumors on CDK4/6. Nevertheless, the results provided evidence for benefits in EWS, malignant peripheral nerve sheath tumors and MB [391]. Of note, within MB subgroups, CDK6 and CDK14 co-amplifications were identified in 20% samples from patients with relapsed group-4 MB [330].

Shubert et al. (2022) also pointed out that patients with atypical rhabdoid tumor/malignant rhabdoid tumor, NB or HGG may also benefit from anti-CDK4/6 therapy [391].

CDK4 and CDK6 are both highly expressed in NB compared to normal tissues [404]. Moreover, like CDK2, CDK4/CDK6 exert oncogenic roles in this tumor type, especially in MYC-amplified forms [405]. Additionally, when co-amplified with MDM2/FRS2, CDK4 and CDK6 are associated with poor prognosis and atypical clinical features, including poorly differentiated or undifferentiated histology and metastasis at diagnosis and at relapse [406].

In line with Schubert et al. (2022) [391], CDK4/6 upregulation also plays an important role in the pathogenesis and progression of high-grade gliomas with potential actionability [407,408,409]. However, the use of CDK4/6 inhibitors alone did not show satisfactory results, suggesting the use of combinatorial intervention [410].

CDK4 was likewise found overexpressed in EPN and associated with adverse outcomes [411]; accordingly, its inhibition restricted cell proliferation and reduced the expression of genes associated with the cell cycle and DNA repair (*CCNB1*, *TOP2A*, *CDK2*, *BRCA1* and *RAD51*), and induced morphological changes that culminated in cell death [412]. Considering EPN subgroups, De Almeida Magalhaes et al. (2020) showed that *CDK6* is overexpressed in ST-EPN-RELA tumors compared to other ST-EPN subgroups [413], even though others have suggested that the dysregulations of the p16-CDK4/6-pRB-E2F pathway might also compose the genetic background underlying the aggressive biology of posterior fossa EPN in infants less than 1 year old [414].

**CDK5**. The cyclin-dependent kinase 5 (CDK5) represents an unusual member of the family of cyclin-dependent kinases, which is activated upon binding to p35 and p39 proteins, which are not cyclins. Conversely, interactions with CCND1 or CCND can attenuate CDK5 activity [415]. CDK5 is expressed ubiquitously, but with higher activity in the nervous system, participating in neuron migration, neurite outgrowth and synaptogenesis. Nevertheless, increasing evidence points to a diverse array of functions in other tissues, ranging from cell proliferation to cytoskeleton remodeling and cell motility by regulating actin dynamics [416,417,418].

Apart from neurodegenerative disorders, amplification and increased expression of CDK5 have been described in multiple tumor types and are associated with worse prognosis and stemness [419,420,421,422,423,424,425,426,427,428,429,430,431]. Mutations located in key domains of CDK5 that influence its structure and post-translational modifications have also been described as contributors to tumorigenesis [432].

The participation of CDK5 in pediatric tumors is purported; however, the stimulation of cancer-related signaling pathways by this kinase remains obscure, and reports are scarce. CDK5 was found to be hyperactivated in NB and its inhibition resulted in cell cycle arrest and morphological differentiation [433,434]. Moreover, as a crucial regulator of neuronal signal transduction, CDK5 can be found differentially expressed in gliomas, progressively augmenting with tumor grade, suggesting an active role not only in tumorigenesis but in aggressiveness as well [424,435,436].

CDK5 also appears to be a central regulator of OS tumorigenesis, with high levels of expression being associated with low survival and increased angiogenesis [437,438]. Interestingly, CDK5 also plays a role in osteoblastic differentiation. Fu et al., for example, demonstrated that CDK5 inhibition promotes the expression of *Runx2*, *ALP*, *OCN* and *OPN* in mesenchymal stem cells, the mineralization of MC-3T3E1 cells and suppresses the migration of the OS cell line MG-63 [439]. Additionally, the CDK5/p35 complex strongly inhibits the Wnt/beta-catenin signaling pathway, also able to stimulate osteoblastic differentiation [440].

The WNT pathway defines a molecular subgroup of MB [441]; thus, it may be assumed that CDK5 might also contribute to this tumor malignancy. In fact, Cdk5 expression has been demonstrated in different MB cell lines and in a reduced cohort of patients; however, its deletion did not alter proliferation, reflecting the more favorable prognosis of MB with WNT activation [441]. Nevertheless, a role for CDK5 in tumor immune evasion through the regulation of PD-L1 was suggested [442].

**CDK7/9**. Cyclin-dependent kinase 7 (CDK7) and 9 (CDK9), apart from directing cell cycle progression, have critical roles in transcription initiation and elongation as regulators of the phosphorylation of the carboxy-terminal domain (CTD) of RNA polymerase II (CDK7 is a component of TFIIH, and CDK9, a subunit of pTEFb) [443,444,445]. CDK7/9 also controls many transcription factors, functioning to either promote their activity and/or regulate their turnover [445]. Recently, other uncovered transcription-associated functions have been revealed, including epigenetic modifications and mRNA-3′ termination [446,447,448].

CDK7/9 levels are elevated in several cancer types and are associated with clinical outcomes [449,450,451,452,453,454,455]. In many cases, they can indirectly impact gene expression profiles by aberrantly controlling the functioning of transcription factors that are critical in specific tumor types, as is the case of estrogen- or androgen receptor-mediated transcription in breast and prostate cancer, respectively [449,456,457]. MYCN-dependent transcription can also be affected, as demonstrated in NB cells, or contribute to histone-3 methylation in diffuse intrinsic pontine glioma (DIPG) [445,458].

With regard to other pediatric tumors, CDK7 has been shown to be upregulated in a panel of OS cell lines and tumor samples, being associated with worse prognosis and higher metastasis rates [459]. Accordingly, CDK7 knockdown in SJSA-1 cells reduced phosphorylation of the RNAPII CTD and reduced tumor volume and weight in xenograft models compared with tumors derived from wild-type cells [460]. Similarly, higher levels of expression of CDK9 have been associated with lower Huvos grade and lower survival rates, characterizing this kinase as a suitable therapeutic target, as determined through siRNA assays [461,462].

Descriptions about the relationship between CDK7 expression and EPN, RMS, EWS, WT, RB and NB are rare. Nevertheless, the use of THZ1 (CDK7 inhibitor) has exposed positive scenarios, considering that EWS cells are sensitive to this compound and that reduced EMT capacity of RB cells is observed after treatment [463,464]. In contrast, CDK9 kinase is widely expressed in RMS, where it impedes the physiological cellular differentiation [448,465,466,467]. Additionally, inhibition of CDK9 demonstrated a general disruption of transcription [465]. Similarly, this kinase is widely expressed in pediatric sarcomas, such as EWS, and its pharmacological inhibition (PHA-767491) enhanced the mithramycin-mediated suppression of the EWS-FLI1 transcriptional program, leading to a shift in the IC_50_ and striking regressions of mouse xenografts. Furthermore, this kinase is upregulated in NB, increasing with the degree of differentiation of the tumor [468]. Of note, Poon et al. (2020) demonstrated that CDK9 inhibitors are able to downregulate MYCN to varying degrees and to induce apoptosis, as detected by induction of poly (ADP-ribose) polymerase (PARP) cleavage [469].

##### Polo-Like Kinases

Polo-like kinases (PLKs) comprise a highly conserved multifunctional family of kinases of five members: PLK1, PLK2, PLK3, PLK4 and PLK5 [470,471,472]. These serine/threonine kinases are traditional controllers of cell cycle progression, with major roles in the formation of the mitotic spindle, chromatid separation, regulation of the anaphase-promoting complex, DNA damage response and cytokinesis [472,473]. Structurally, these proteins share an N-terminal highly conserved catalytic domain and a regulatory domain fundamental to the functionality and localization of PLKs, called Polo-Box (PBD) and located at the C-terminus [474].

PLKs are differentially expressed depending on the tissue and cell cycle phase [475]. Alterations in the expression of PLK genes have already been described in different types of cancer (breast, OS, leukemia, gliomas, among others) and have generally been correlated with dismal prognosis [476].

**PLK1.** PLK1 is the most studied member of the family. This protein plays key roles at different points of the cell cycle, especially during the progression of mitosis [477]. Nevertheless, other non-mitotic functions such as cell survival, genomic maintenance, cell fate and DNA damage control are also regulated by PLK1 through the interaction of with effector pathway components, including the oncogenes AKT, MYC, MDM2, Β-catenin and the tumor suppressors P53, PRB, BRCA2 and PTEN [478,479].

A plethora of studies have firmly established the active role of this kinase in oncogenesis and its prognostic value along with its potentiality as a therapeutic target [480,481,482,483,484,485,486]. Childhood cancer is not an exception. Higher levels of PLK1 have been observed in a variety of cell lines, including EWS, OS, NB and RMS [483,484]. Moreover, this protein has been described as overexpressed in MB samples, where it is associated with higher recurrence and lower survival rates [487,488]. Furthermore, other studies have validated this positive correlation between PLK1 expression and higher cell proliferation in, mainly in undifferentiated tumors with the presence of massive choroidal invasion [205,476,489,490,491]. PLK1 overexpression is also present in unfavorable NB and associated with poor prognostic markers such as lower age at diagnosis and MYC amplification [492]. This interaction between PLK1 and MYC has also been observed in OS, in which the kinase contributes to MYC stabilization [493].

##### Aurora Kinases

Three members of the Aurora family of serine/threonine kinases have been identified in humans: Aurora kinase A (AURKA), Aurora kinase B (AURKB) and Aurora kinase C (AURKC). These kinases (named after the resemblance or their localization to the poles of the mitotic spindle to the way aurora borealis are observed at one of the poles of the earth) have pivotal parts in the execution of mitosis (AURKA and AURKB) and meiosis (AURKC), and even exerting conserved function, they cannot fully compensate for the loss of one another [494,495].

**AURKA.** Aurora kinase A is involved in the centrosome maturation process and promotes the transition from G2 to mitosis. AURKA levels increase along late S and G2 phases and reach a higher peak in mitosis, followed by proteasome-dependent degradation [496,497]. This kinase is early localized at the centrosome and regulates the progression of mitosis by phosphorylation of multiple substrates, promoting mitotic entry through the activation of Cyclin-B/CDK1 [498]. Moreover, AURKA progressively associates with the mitotic poles and the adjacent spindle microtubules, contributing to chromosome separation and bipolar spindle [499,500].

Among the three human aurora kinases, AURKA has been the family member most consistently associated with cancer. Amplification of the chromosomal region 20q13 where the AURKA gene is located is commonly observed in cancer cells [501,502,503]. Nevertheless, according to Mou et al., almost 90% of tumors present in the TCGA database show AURKA overexpression [504]. Indeed, high levels of AURKA expression can endorse abnormal cell cycle progression, resulting in genomic and chromosomal instabilities, which are hallmarks of highly proliferative tumors [505,506]. Thus, AURKA expression not only enhances proliferation, but may also influence other processes, including apoptosis evasion, EMT, drug resistance and metastasis [507,508,509,510,511,512,513,514,515,516,517].

Besides AURKA mitotic functions, other non-canonical and kinase-independent activities have been gradually discovered in cancer cells. After mitosis, most AURKA proteins degraded, but a remnant population may be still detected inside the nucleus, pointing to the possibility that the kinase could work as a transcriptional regulator [518]. In this regard, AURKA overexpression has been associated with the upregulation of stem cell markers such as SOX2 and NANOG, imposing participation in the maintenance of the self-renewal capacity of cancer stem cells (CSCs) [519].

In pediatric tumors, AURKA polymorphism rs8173 G > C has shown to decrease WT risk [520]. Conversely, AURKA plays an active role protecting MYCN from ubiquitinylation and proteolysis in NB, thus contributing to more aggressive phenotypes and poor survival probability [521,522]. Moreover, this role has also been described in RMS, where AURKA not only stabilizes MYC but also PAX3-FOXO1 [523]. Moreover, in this tumor type, AURK is overexpressed and considered a key factor in the observed aneuploidy and chromosomal instability [524]. AURKA is also closely related to the oncogenic process of EWS and is considered a chemotherapy resistance and may act as a potential biomarker for prognosis [525,526].

AURKA overexpression has also been associated with OS, as many cell lines are highly sensitive to its inhibition [527,528]. Similar patterns have been seen for WT, where AURKA inhibition impaired tumor growth and induced apoptosis both in vitro and in vivo; however, such effects were improved in RB1-deficient cell lines compared to those with *MYC* amplification [529].

Moreover, expression profiling of pediatric brain tumors has shown that AURKA was consistently and highly overexpressed (up to 106-fold) in tumor samples from all glioma grades and from patients varying from 4 months to 82 years old; however, mRNA expression showed only weak correlation with the Ki-67 labeling index, and significant associations with poor patient survival were only observed for GBM [530]. Overexpression of AURKA is also linked with survival in MB patients [531].

**AURKB.** The second member of the family, Aurora kinase B (AURKB) is one of the most intensively studied kinases because it provides catalytic activity to the chromosome passenger complex (CPC), formed by AURKB, INCEP (inner centromere protein), survivin and borealin (Cell 2002;13:3064–77). The CPC governs highly different processes, such as chromosome alignment, histone modification and cytokinesis [532,533,534,535]. Additionally, AURKB kinase activity is essential for faithful chromosome segregation and functions to correct any improper kinetochore attachment to the spindle [532,536,537,538]. Finally, Aurora kinase B (AURKB) is also essential in mitotic DNA damage response, protecting against DNA damage-induced chromosome segregation errors, including the control of abscission checkpoint and prevention of micronuclei formation [539]. Consequently, AURKB dysregulation results in aneuploidy and genomic instability and in turn promotes cell cycle progression and survival of cancer cells [540,541,542,543]. Indeed, expanding evidence at the gene, mRNA and protein levels supports a carcinogenic role of AURKB. Overexpression has been reported in clear cell renal cell carcinoma (RCC) and cervical carcinoma, among many others, with clear associations with clinicopathological parameters such as stage and tumor volume, chemoresistance, tumor progression and poorer survival [544,545,546,547,548,549,550,551,552,553].

In the pediatric setting, overexpression of AURKB was closely correlated with poor prognosis and carboplatin resistance in NB patients [552,554]. Similar profiles were observed for pediatric ALL and AML patients, especially in T-cell and E2A-PBX1-translocated ALL cases. Further in vitro assays demonstrated that AURKB is an essential protein for the proliferation and survival of acute leukemia cells [555].

The importance of Aurora kinases as potential therapeutic targets for childhood brain malignancies is highlighted by AURKB being highly and consistently overexpressed in the majority of high-grade gliomas, but despite reflecting the presence of aneuploidy, at least in EPN, it did not emerge as a prognostic factor [556,557]. For other tumor types, however, data about the prognostic value of AURKB are less explored and primarily rely on experimental assays using pharmacological inhibitors. In this context, OS, EWS and RB are included [558,559,560].

**AURKC.** Differentially from AURKA and AURKB, Aurora kinase C (AURKC) is limited to cells that undergo meiosis (sperm and oocyte). This kinase is located on human chromosome 19q13.43, regulated by promoter methylation and when expressed in germ cells, can undergo alternative splicing resulting in three protein variants [561,562,563]. As the major enzymatic component of the CPC during meiosis, it plays a specific role during human female meiosis and preimplantation embryo development [564].

A body of evidence shows that overexpression of AURKC in mitotic cells leads to centrosome amplification and multinucleation [565]. Its upregulation and other CPC components occur in cancer cells and may correlate with clinical characteristics [566,567]. In line with this, AURKC is overexpressed in tumors of the reproductive system and in breast and prostate cancer cell lines [568,569]. Nevertheless, varying degrees of CpG islands hypermethylation leading to lower AURKC mRNA levels have been described in WT, suggesting that this kinase might not be of importance in this childhood tumor [570]. Likewise, AURKC expression was not associated with survival or risk status in neuroblastoma patients [571]. In OS, knockout of AURKC displayed no changes in cell proliferation, migrated less and formed fewer colonies in soft agar compared to wild-type cells. Moreover, whole-transcriptome sequencing revealed over 400 differentially expressed genes which included genes encoding proteinaceous extracellular matrix components, suggesting that therapeutics targeting this aurora kinase isoform could decrease cancer cell metastasis and disease progression, the most limiting characteristic of survival [572].

### 3.2. In Silico Analysis of Different Kinases Expression and Their Association with Clinical Prognosis

**Figure 3:**
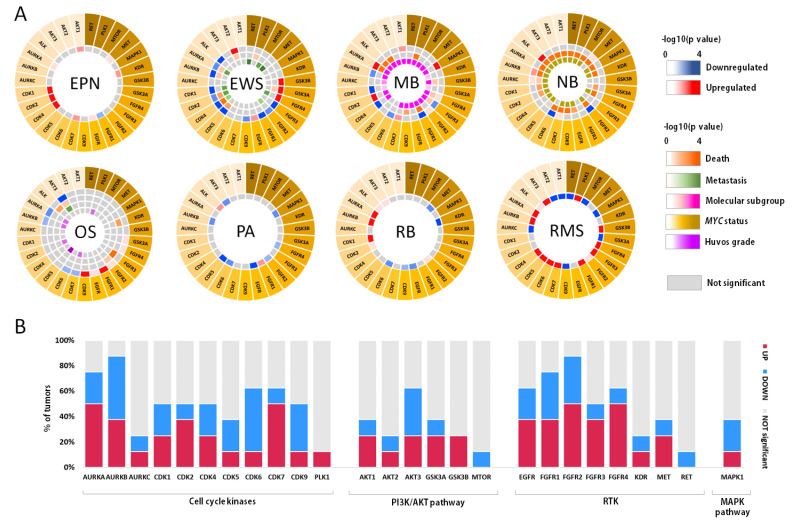
(**A**) Polar plots of differentially expressed kinases in pediatric tumors obtained through the analysis of available data on the R2: Genomics Analysis and Visualization Platform (http://r2.amc.nl (accessed on 15 October 2022)). Tumor abbreviations: EPN—ependymoma; EWS—Ewing sarcoma; MB—medulloblastoma; NB—neuroblastoma; OS—osteosarcoma; PA—pilocytic astrocytoma; RB—retinoblastoma; RMS—rhabdomyosarcoma. *p*-values are represented by differential coloring gradients. The external inner circle corresponds with “tumor versus normal tissue” results. The other concentric layers represent data related to associations with clinical features: metastasis, death, molecular subgroup (MB), MYC status (NB) and Huvos grade (OS). For actual *p*-values, refer to Supplementary Table S2. (**B**) Percentage of tumors with altered expression of each kinase. Few commonalities were found.

According to our systematic in silico analysis of the selected group of kinases, comparisons of expression patterns between pediatric tumors and normal samples showed varying results, and despite what was expected from the data already published, few commonalities were found (Figure 3A,B, Supplementary Table S1). Considering CNS tumors, overexpression of *AKT1*, *AURKA*, *CDK2* and *CDK7* was observed in EPN and MB, but not in PA. Similarly, EPN showed higher levels of C*DK1*, *CDK4*, *EGFR*, *KDR* and *MET*, while for MB, the upregulation of *FGFR2*, *FGFR4* and *MAPK1* was highlighted. Conversely, EPN samples demonstrated low levels AKT3 and FGFR1, while CDK*1/4/6*, *FGFR1/3* and AURKB were downregulated in MB tissues. PA, on the other hand, exhibited high levels of only *ALK* and *FGFR1*, and downregulated genes included *AKT3*, *AURKB*, *CDK5*, *EGFR*, *FGFR2*, *FGFR4* and *MAPK1*.

Neuroblastoma was the tumor type with the more reduced number of hub genes. In this tumor type, CDK6 and FGFR2 were less expressed than in normal tissue, whereas *CDK7*, *MET*, *ALK* and *AURKB* stood out as upregulated in tumor samples. Concomitantly, RB showed high levels of *AKT3*, *ALK*, *AURKA*, *AURKB*, *CDK1*, *CDK2* and *FGFR2* genes and low levels of CDK6, CDK9, EGFR, KDR and MET.

Among sarcomas, RMS was the tumor type with the most altered kinase profile, including high levels of *AKT3*, *ALK*, *AURKA*, *AURKB*, *CDK4*, *CDK5*, *CDK6*, *CDK7*, *GSK3B*, *PLK1* and all *FGFR*. Then, *AKT2*, *EGFR*, *FGFR4* and *GSK3s* (A and B) showed higher expression in EWS, contrasting the low levels of *ALK*, *AURK*s (A and B), *CDK*s (1, 2, 4, 5 and 9) and *FGFRs* (1, 2 and 3). Finally, our analysis of OS samples demonstrated upregulation of the receptor genes *EGFR* and *FGFR* (1, 2 and 4), as well as *CDK9* and *GSK3A*. Alternatively, *CDK6/7*, *AURKA/B* and *AKT3* had low expression profiles.

Notwithstanding, further analysis showed that, in the same line as reported in the literature, the expression of most of the selected kinases is indeed associated with clinical features of worse prognosis, including associations with MYC amplification in NB and molecular subtypes in MB, and metastases in bone sarcomas (Supplementary Table S2).

## 4. Kinases as Druggable Targets—Evidence and Limitations

The gradual advancements in genetics and biochemistry during the second half of the last century not only contributed to the better understanding of molecular events underneath signaling pathways in both natural and pathological settings, but also laid the foundation for the development of modern targeted agents. Perhaps the most expressive example of that trajectory involves chronic myeloid leukemia and the “Philadelphia chromosome”. After its simple description (250 words) by Nowell and Hungerford, it took a decade to properly identify the chromosome pairs involved in the translocation [573]. It was only after the introduction of the G-bands by Marina Seabright that Janet D. Rowley from the University of Chicago that it was possible to identify the little chromosome as a result of the reciprocal translocation between chromosomes 9 and 22, specifically, t(9;22)(q34;q11) [574,575]. However, its molecular characterization only came to light between 1982 and 1984 [576,577,578], demonstrating the in-frame juxtaposition of the *ABL* oncogene (on chromosome 9) with the *BCR* gene (on chromosome 22), resulting in the hybrid *BCR/ABL* gene that gives rise to a chimeric protein with high tyrosine kinase activity and with a critical role in the development of leukemia [579]. Later, the discovery of this tumor-specific protein led to development of imatinib mesylate, providing an incredibly successful treatment that converted a fatal cancer into a manageable chronic condition, and pioneered an era of target-directed therapy [580].

More recently, the emergence of integrative laboratorial methods such as kinome-wide siRNA screens, next-generation sequencing (NGS) and phosphoproteomics have dramatically intensified the assortment of kinase inhibitors for the treatment of human cancers, currently accounting for about a quarter of all drug discovery research and development efforts.

Moreover, the increasing number of databases and analytical and visualization tools has facilitated advanced drug discovery not only by gathering information about the prognostic value of specific genes in oncology, but also it is now possible to access chemical structures and docking, affinities and structural features of approved small-molecule inhibitors in more easily, accessible and systematic ways, thus accelerating the discovery and optimizing screening to more direct translational assays.

In this regard, to further illustrate the importance of the selected kinases’ dysregulation in the pathophysiology of pediatric cancer, other bioinformatic tools were used (Supplementary Figure S2). As a first step, we analyzed the vulnerability of different cancer cell lines against their inhibition through the Cancer Dependency Map portal (http://depmap.org accessed on 14 December 2022), a platform that provides information about how dependent different cell lines are on a specific gene depletion based on CRISPR and RNAi knockout experiments. The results are presented as a score generated by the platform itself: greater than zero (>0) indicates that the cell line is not dependent, less than zero (<0) indicates that the lineage is dependent and scores below −1 indicate that the analyzed gene is essential for the survival of the cell lineage.

**Figure 4:**
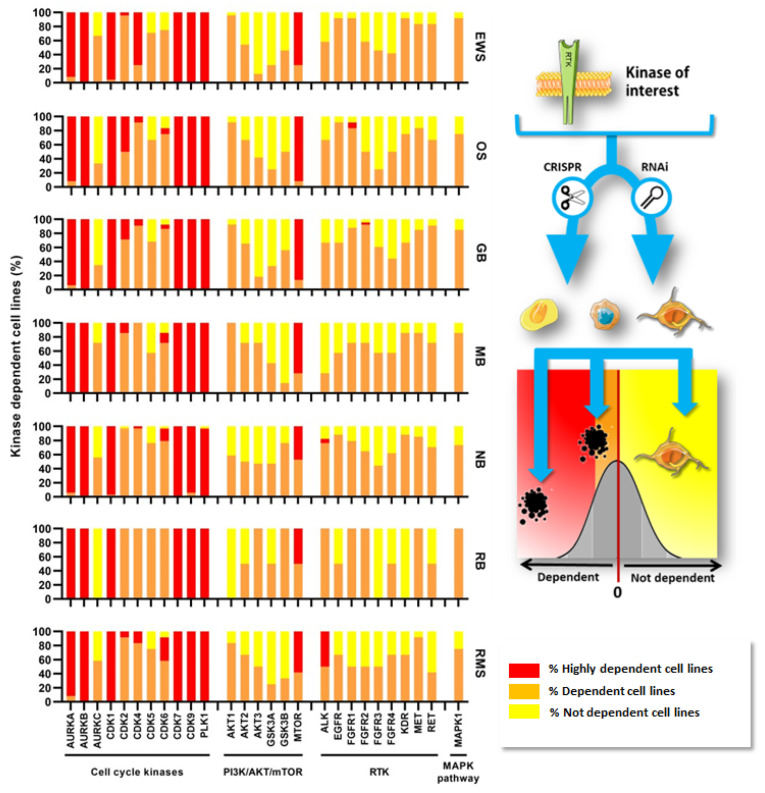
Percentage of pediatric cell lines dependent on the selected group of kinases. Dependency data were imported from the DepMap consortium (CRISPR (DepMap 22Q2 Public + Score, Chronos; https://depmap.org/ portal/ (accessed on 30 October 2022)) and classified as highly dependent, dependent or not dependent. The data were plotted on a histogram where it is possible to see the vulnerability of pediatric cell lines mainly to cell cycle kinases and PI3K/AKT/mTOR families. Cell lines selected included Ewing sarcoma (EWS), osteosarcoma (OS), glioma (GB), medulloblastoma (MB), neuroblastoma (NB), neuroblastoma (NB), retinoblastoma (RB) and rhabdomyosarcoma (RMS). Dependency scores for each cell line are shown in Supplementary Table S3.

Initial screening showed that all or most cell lines are dependent on the kinases analyzed, with comparable scores between adult or pediatric origins (Figure 4; Supplementary Table S3). Interestingly, a similar pattern occurs across the different tumors, irrespective of histology. Almost 100% of the cell lines are highly dependent on cell cycle kinases, especially AURKA/B AURKB, cyclin-dependent kinases CDK1 and CDK7/9, and PLK1. Cell lines were also highly dependent on mTOR. Conversely, cell lines were less vulnerable to the depletion of AURKC, AKT3, GSK3A and FGFR3, with more than 50% of cell lines presenting scores above 1 (in line with published data reviewed above).

**Figure 5:**
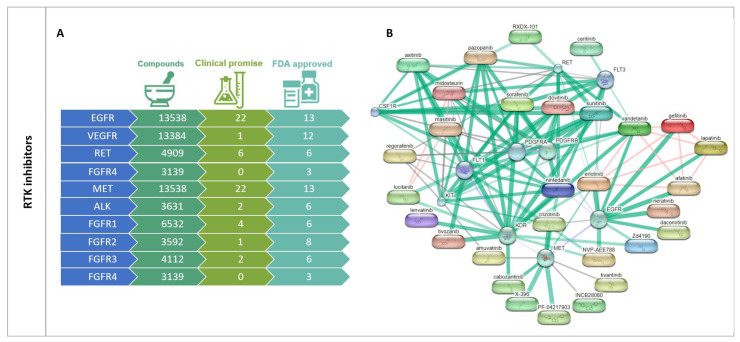
(**A**) Schematic illustrations of RTK druggability identified by the CanSAR database, including the total number of compounds with predicted interaction capacity with each kinase, as well as FDA-approved drugs and clinical candidates. (**B**) Interaction networks of RTK inhibitors and associated binding proteins according to STITCH (search tool for known and predicted interactions between chemicals and proteins available at http://stitch.embl.de (accessed on 1 November 2022)). Compounds are represented as pill-shaped nodes, while proteins are shown as spheres. Small nodes represent proteins of unknown 3D structures, while large nodes show proteins with known or predicted structures. Nodes that are associated with each other are linked by an edge: thicker lines represent stronger binding affinities. Networks were constructed considering a minimum required interaction score of 0.700, and based on associations reported in curated databases (gray lines), or on both databases and experimental/biochemical data (green lines). Purple lines represent functional links between proteins.

Then, aiming to further exemplify information on individual kinase-targeted compounds and their analogs, we performed a search through the CanSAR knowledgebase (http://cansar.icr.ac.uk accessed on 14 December 2022), an integrative platform that compiles multidisciplinary data and provides useful drug discovery predictions. The analysis of predicted compounds for our selected group of kinases showed more than 55,000 potential compounds that are able to target RTK, with EGFR and VEGFR representing the most druggable ones. As shown in Figure 5, FDA-approved drugs are already identified for all the RTKs, and more than 20 additional drugs are being studied as novel clinical candidates.

For PI3K, although the CanSAR analysis revealed more than 20,000 potential compounds, only the mTOR inhibitors perhexiline and everolimus are described as FDA-approved drugs. Nonetheless, 15, 4 and 2 clinical candidates are being investigated as specific inhibitors for mTOR, AKT1 and GSK3B, respectively (Supplementary Figure S3). Approved drugs for MAPK are also scarce, including only sorafenib, regorafenib, dabrafenib and encorafenib, all of which target RAF1. For this specific kinase, CanSAR identified 4764 promising compounds and three clinical candidates (Supplementary Figure S3). Among the cell cycle kinases, CanSAR identified only approved drugs targeting CDK4 and CDK6. However, many clinical candidates (more than 15) targeting the other kinases of this group are being studied. According to the platform, cell cycle kinases are the second druggable category of kinases with the highest number of compounds described as potential specific drugs, totaling more than 48,000 (Supplementary Figure S3).

Further, for compilation of the preclinical results on kinase inhibitors, and considering that information in the literature often appears scattered and fragmented, the following section shows published evidence for (1) solid tumors that showed differential gene expression through in silico analysis (refer to Figure 3), (2) compounds considered “FDA approved” or with “clinical promise” and (3) compounds that have been assessed in vitro or in vivo before entering clinical trials. Thus, in the following subsections, experimental data on individual kinase inhibitors are detailed within each category. For further information, see Supplementary Table S4.

### 4.1. RTK Inhibitors

**Erlotinib (Tarceva^®^).** This compound is a quinazoline derivative that selectively and reversibly inhibits EGFR [581]. Erlotinib is an FDA-approved drug for the treatment of NSCLC and pancreatic cancer in combination with gemcitabine chemotherapy [582]. In the pediatric setting, this inhibitor has shown contrasting results. As monotherapy, Erlotinib was not efficient in reducing cell growth in a panel of NB cell lines, albeit effective indirect responses were obtained in xenograft tumors [583]. Similar results were obtained in sarcomas. This compound alone was ineffective in OS cells, in which the STAT3 cascade pathway has been pointed as the molecular mediator of both intrinsic and acquired resistance. In EWS, this compound alone or in combination did not inhibit growth of tumor xenografts and even led to a decrease in the therapeutic activity of cyclophosphamide when compared to single-agent activity [584,585]. Erlotinib had no effect on tumor progression in genetically engineered ARMS mouse models [586].

In contrast, satisfactory results were obtained in CNS tumors, where erlotinib therapy inhibited MB migration in vitro and successfully diminished the levels of phosphorylated EGFR in EPN models [587,588]. Treatment with this drug was also cytotoxic in Y79 and WERI RB cells in a dose-dependent manner, leading to cell cycle arrest and reduced migration, while oral administration dramatically reduced the growth of Y79 tumor grafts [589]. Regarding patients, phase I clinical studies have been developed in order to evaluate the acceptable tolerability profile in cases of brain and refractory solid tumors including RMS, soft tissue sarcomas, NB or germ cell tumor. Children appeared to tolerate erlotinib similarly to adult patients, and drug disposition was similar between these populations. The combination of temozolomide and erlotinib was well tolerated and it was also suggested in combination with radiotherapy [590,591].

**Vandetanib (Caprelsa^®^).** This is a multitargeted tyrosine kinase inhibitor with potent effects against VEGFR2/3, EGFR and RET [592,593]. This compound is approved to treat medullary thyroid cancer that cannot be removed by surgery and is locally advanced or has metastasized, and has demonstrated modest efficacy in patients with metastatic breast cancer [594,595]. Regarding pediatric tumors, vandetanib has been shown to inhibit the proliferation of NB cells mediated by the induction of G1-phase cell cycle arrest at lower concentrations and by apoptosis at higher concentrations. Migration and invasion were also markedly decreased compared with the control group [596,597]. Treatment also decreased (p)RET expression in five other NB cell lines and strongly impaired tumor growth in vivo in both MYCN/KI AlkR1279Q and MYCN/KI AlkF1178L mice, and was able to sensitize cisplatin-resistant NB subcutaneous tumor growth with less severe liver toxicity compared with high-dose cisplatin [598,599,600]. Moreover, vandetanib, in combination with 13-cis-retinoic acid, reduced tumor vascularity and induced apoptosis in NB xenografts [601]. Indeed, according to Craveiro et al. (2017), the narrow target spectrum of Vandetanib along with a favorable toxicity profile makes this drug ideal for multimodal treatment approaches. These authors tested this compound against SHH-TP53-mutated and MYC-amplified MB cell lines and found that it leads to a dose-dependent reduction in cell viability, interferes with clonogenicity and has pro-apoptotic effects after 48 h. Of note, combinations with GDC-0941 (clinically available PI3K inhibitor) and etoposide resulted in complete loss of cell viability [602]. The combination of vandetanib and celecoxib displayed a synergistic or additive antitumor effect on OS in vitro and in vivo [603]. However, combinations of gefitinib and vandetanib only inhibited the proliferation of EWS cell lines at very high concentrations (>1 μM vandetanib, >5 μM gefitinib), indicating the action on off-target effects [604].

**Gefitinib (Iressa^®^).** This drug, also known as ZD1839, is a member of the 4-anilinoquinazoline class of compounds that specifically and selectively inhibits EGFR [605]. In preclinical studies, gefitinib treatment was associated with growth inhibition and increased apoptosis in human cancer cell lines, and antitumor effects against xenografts of human tumors [606,607]. Gefitinib was shown to inhibit proliferation in juvenile PA primary cell cultures with an IC_50_ determined between 1.6 and 9.6 µM [608]. In addition, this compound was shown to inhibit invasion and metastasis of intratibial OS xenografts via inhibition of macrophage receptor interacting serine-threonine kinase 2 (RIPK2) [609]. Moreover, in children, Gefitinib has been tested for refractory solid tumors and CNS malignancies, showing similar pharmacokinetics as in adults [610].

**Regorafenib (Stivarga^®^).** This drug is an oral multikinase inhibitor that targets VEGFR1/3, FGFR and other receptor kinases [611]. This compound is already approved to treat metastatic cases of colorectal cancer and advanced hepatocellular carcinoma (HCC) previously treated with Sorafenib [610]. In vitro, regorafenib exhibits antiproliferative effects against a panel of 33 pediatric tumor cell lines, including MB (D341 Med, Med-Meb-8A), OS (IOR-OS-18), EWS (EW7, ORS, POE, SIM, STA-ET-1) NB and (SJ-NB-8, SK-N-BE(2), SH-SY5Y), with a mean half maximal growth inhibition of 12.5 μmol/L [612]. Particularly in the last, Regorafenib has shown to be effective against through the inhibition of RAS/MAPK, PI3K/Akt/mTOR and Fos/Jun pathways [612]. Similarly, regorafenib demonstrated antitumor activity in animals bearing subcutaneous RMS, EWS (STA-ET-1 and EW7) and NB (SJ-N-B8 and SK-N-AS) xenografts with tumor growth inhibition ranging from 73% to 93%. Moreover, when associated with radiation and irinotecan, it induced 100% regression in an MB patient-derived xenografts (PDX) model [610].

**Dacomitinib (Vizimpro^®^).** Also known as PF-00299804, this drug is an orally administered, second-generation, irreversible inhibitor of EGFR, HER2 and HER4, which has shown positive anticancer activities in some preclinical and clinical trials, being approved by the FDA for the treatment of metastatic NSCLC [613,614]. Besides its ATP-competitive action, dacomitinib covalently binds to Cys773 located in the ATP-binding cleft of EGFR, which irreversibly blocks ATP binding and inactivates the receptor [615]. For pediatric MB, dacomitinib has shown to block EGFR/HER signaling in DAOY cells and in orthotopic xenografts, extending median survival as a single agent; however, it was antagonistic when used in combination with standard frontline chemotherapy (4HPC, vincristine or cisplatin) [616].

**Lapatinib (Tykerb^®^).** This compound is an oral dual tyrosine kinase inhibitor that inhibits human EGFR and blocks the EGF receptor 2 (HER2) [617]. It was FDA-approved to treat HER2-positive advanced or metastatic breast cancer as monotherapy or in combination with other drugs [618]. Lapatinib has been tested in childhood solid tumors (including RMS, EWS and NB) and leukemia cells by the NCI-supported Pediatric Preclinical Testing Program (PPTP) [619]. In this study, among 23 cell lines, fifteen achieved at least 50% growth inhibition, and the median IC_50_ value for lapatinib against the entire cell line panel was 6.84 μM (range 2.08 μM to >10.0 μM). In vivo, however, lapatinib presented little activity against the 41 xenograft models of pediatric tumors [619,620].

**Cetuximab (Erbitux^®^).** This compound, available as Erbitux^®^ (Merck Sereno), is a human–murine chimeric monoclonal antibody that competes to bind to the extracellular domain of EGFR and has been approved for the treatment of colorectal and head and neck cancer [620]. Information about preclinical use of this compound is scarce. However, a report showed that the proliferation of RMS cell lines was not influenced by this EGFR inhibitor [621]. However, later, it was shown that the combination of cetuximab and actinomycin D was highly effective in EGFR-positive RMS cells (RD and Rh30, of embryonal and alveolar origin, respectively), synergistically inhibiting cell growth and inducing apoptosis [622].

**Sunitinib:** Sold under the brand name Sutent^®^, this drug is a small-molecule multitarget inhibitor functioning on PDGFR, VEGFR, KIT, Flt-3 and RET [623,624,625]. In a preclinical study, this drug demonstrated limited growth inhibitory effects in a panel of 23 pediatric cell lines that included OS, ALL, EWS, RMS, MB, EPN, NR, GBM, WT and others [625]. However, in vivo, it presented growth inhibitory activity against pediatric solid xenograft models of EWS, RMS and NB [625]. A later study showed decreased cell proliferation and phosphorylation of VEGFRs NB cells after treatment with sunitinib, and tumor growth, angiogenesis and metastasis in tumor xenograft models [624]. Moreover, in combination with an mTOR inhibitor (rapamycin), it showed a synergic cytotoxic effect, which was more effective than the traditional chemotherapeutic agent cyclophosphamide [624].

**Lenvatinib (Lenvima^®^).** This drug is a synthetic, orally available type I tyrosine kinase inhibitor exhibiting powerful antiangiogenic activity currently used to treat certain types of thyroid cancer and potentially other tumor types [626]. Lenvatinib was initially reported in 2008 as a multitargeted RTK inhibitor of VEGFR1/2/3, but it also inhibits FGFR1–4, PDGFR-alpha and KIT [627,628,629]. Preclinical findings in sarcomas indicated that lenvatinib was able to inhibit tumor growth in xenografts obtained through direct implantation of patient tumor specimens in nude mice. The experiment showed positive results in 7 out of 10 xenografts accompanied by marked decrease in microvessel densities. However, in vitro, Lenvatinib did not show potent effects on tumor viability in OS-derived cell lines [629]. Others showed that the drug was able to inhibit tumor cell migration and invasion in U2OS cells [630]. Further, in a phase I/II study, lenvatinib as a single-agent reported a response rate of 7% and a median progression-free survival of 3 months in a cohort of 31 children and young adults with OS, although many patients had treatment-related adverse events of grade ≥3 [631]. In other pediatric tumors, the effects of lenvatinib remain to be investigated.

**Pazopanib (Votrient^®^).** This compound is an FDA-approved pan-VGFR inhibitor, even though it also targets PDGFR-α and -β, FGFR1/3, KIT as well as BRAF proteins [632]. In a pan-cancer study, pazopanib was unable to affect the viability of the any of the treated cell lines, which included SK-N-BE(2) (N-Myc amplified) and SH-SY5Y (non-N-Myc amplified) NB cell lines, the KHOS OS cell line, and the RMS cell lines RH30 and RD. However, in combination with topotecan, this compound showed significant antitumor activity in vitro and halted tumor growth in NB xenograft-bearing mice, but after 50 days, gradual growth was observed [633,634]. The combination of pazopanib with trametinib showed antitumor effects in vitro and in vivo against a panel of seven OS cell lines, in which treatment reduced proliferation and colony-forming capacity and increased the percentage of apoptotic and dead cells. In MNNG/HOS and KHOS xenograft models, both drugs induced a significant inhibition of tumor growth compared to the untreated controls [635]. The in vivo antitumor activity of pazopanib was also tested by the PPTP Program in a subset of sarcoma models that also included EWS and RMS. Although objective responses were not observed for any of the sarcoma xenografts studied, treatment prolonged survival [636]. Even with modest benefits, pazopanib has been approved for line treatment of metastatic non-adipocytic soft tissue sarcomas after the failure of standard chemotherapy. Its efficacy in patients with OS is limited to case reports [637]. One metastatic extraosseous EWS was also reported as successful after treatment with pazopanib [638].

Regarding CNS tumors, EPN cells showed to be sensitive to Pazopanib with a viability reduction of around 35% at 1 μmol/L [639]. Additionally, treatment with Pazopanib reduced the mobility of MB cell lines, inducing clumping of the actin microfilaments (which facilitated cell detachment), as detected by wound healing assays and Fluor-555-coupled phalloidin [640]. Further in vivo tests demonstrated delayed growth of group-3-MB cells transplanted into the cerebellum of mice and prolonged survival (by 10 days) of mice treated once daily by gavage with 60 mg/kg compared to untreated controls [641]. Alternatively, for patients with recurrent high-grade gliomas as part of phase I or II clinical trials, this drug has not been beneficial [642].

**Cabozantinib (Cometriq^®^).** This compound is an orally available multitarget tyrosine kinase inhibitor that inhibits VEGFR1/2/3, MET, KIT, RET, AXL and FLT3. FDA-approved since 2012, it is currently used to treat metastatic medullary thyroid cancer, RCC, HCC and differentiated thyroid cancer [643,644]. In preclinical studies, reports of its anticancer effects include the inhibition of metastasis, angiogenesis and tumor growth [645,646,647]. In vitro, cabozantinib has been shown to diminish the cell viability of EWS and OS cells in a dose-dependent manner [160]. Moreover, it also interferes with migration and the microenvironment by inducing the production of osteoprotegerin and causing a decrease in the synthesis of the RANK ligand by osteoblasts [648]. Positive effects on decreasing proliferation were also observed in MB with no differences between cell lines corresponding to different molecular subgroups [649]. Cabozantinib also exhibited anti-proliferative effects in NB cells and reduced cell migration in vitro and significantly inhibited tumor growth of orthotopic xenografts on a daily basis [650].

**Nintedanib (Ofev^®^).** This drug, commercially available under the brand names Ofev and Vargatefi, is an indolinone-derived inhibitor of multiple kinases including VEGFR, FGFR and PDGFR. Recently approved for the treatment of idiopathic pulmonary fibrosis and advanced non-small cell cancer of adenocarcinoma tumor histology, it exerts its antitumoral activity by reducing proliferation, migration and angiogenesis [651,652,653]. Considering pediatric tumors, nintedanib has been shown to inhibit growth in EWS (A673, CHP100) and OS (SaOS2) cell lines, with a key role in controlling OS lung metastatic growth by blocking the fibrogenic reprogramming of OS stem cells (OSCs) [654,655]. Growth inhibition was also observed in a panel of 13 RMS cells, with the PAX3–FOXO1 fusion-gene-positive ones more sensitive to treatment [656]. Moreover, there are reports of EPN cells being sensitive to nintedanib treatment, while this drug is able to extend the survival of mice bearing ST-RELA xenografts [657,658].

**Midostaurin** (Rydapt/Tauritmo^®^). Also known as PKC412 and CGP 41251, this small molecule acts as a multikinase inhibitor targeting PKCα/β/γ, Syk, Flk-1, Akt, PKA, c-Kit, c-Fgr, c-Src, FLT3, PDFRβ and VEGFR1/2. Presenting anticancer roles in vitro and in vivo, it is currently approved for the treatment of acute myeloid leukemia and advanced systemic mastocytosis [659,660]. Midostaurin has been shown to be an efficient anti-sarcoma agent. Indeed, it inhibited EWS cell proliferation in a dose- and time-dependent manner and decreased tumor growth in vivo [661,662]. Moreover, the combination of midostaurin with the cytokine oncostatin M has been shown to be efficient in reducing in vivo tumors, pointing to this combination as a potential adjuvant treatment for OS [663].

**Axitinib (Inlyta^®^).** Also known as AG-013736 this is an oral VGEFR1/3 and PDGFR inhibitor explored to control angiogenesis [664]. Currently, this compound is approved for treatment as monotherapy or in combination with other drugs for renal carcinoma, and is under phase I, II and III clinical trials for many other tumor types [664]. Pre-clinical studies in EPN showed that this drug inhibited PDGFRα and PDGFRβ, and reduced the expression of mitosis-related genes including *ASF1B*, *MKI67*, *HMGA1*, *BRCA2*, *ESPL1*, *TACC3*, *CDC25A*, *RAD51AP1*, *AURKA*, *BUB1B*, *CENPE* and *HELLS*. It also decreased proliferation resulting from cellular senescence [639]. Similar antiproliferative effects were observed in MB 2D and 3D cell cultures, without affecting normal brain cells. Of note, the compound efficiently crossed the blood–brain barrier (BBB), reducing growth rates of experimental brain tumors without acute toxicity in juvenile rats [649]. In GBM, the cytotoxic activity of Axitinib was also reported in vitro and in vivo, characterized by an anti-angiogenic effect and survival prolongation [665]. Moreover, combinations of axitinib and other therapeutic targets have been explored with satisfactory results [666]. Indeed, the combinatorial treatment of Axitinib and PLK4 inhibitor has shown to be beneficial in MB and RMS [667]. Combinations with etoposide or gemcitabine also showed favorable effects on preventing tumor progression in an orthotopic group-3-MB xenograft models [649,668]. Furthermore, in immunodeficient and immunocompetent orthotopic GBM models, axitinib + G47Δ-mIL12 resulted in an extensive decrease in vascularity, increased macrophage infiltration and significant tumor necrosis [669]. Such a antimetastatic effect was also observed in NB [670].

**Ramucirumab (Cyramza^®^).** This is a humanized monoclonal antibody that acts by binding to VEGFR-2, thus limiting angiogenesis and the proliferation and migration of human endothelial cells [671]. Preclinical studies in NB, RB, OS, RMS and EWS have also shown that ramacirumab enhances anti-tumor activity by abrogating endothelial cord formation, while in vivo, it has also induced tumor growth delay. However, modest or no response was observed in OS [672]. This compound was approved by the FDA in 2014 and indicated for the treatment of gastric cancer, NSCLC, colorectal cancer and HCC [673,674,675]. As a well-tolerated drug, its combinatorial use was also approved, even though its use in clinics is limited due to a lack of specific markers and high costs [676].

**Alectinib (Alecensa^®^)**. Also known as CH5424802, this is an orally available selective ALK inhibitor already approved by the FDA for lung cancer treatment [677]. The compound is able to bind wild-type ALK and its fusions and its anticancer effects have been widely described. Noteworthy, it has shown acceptable results after treatment of intracranial EML4-ALK-positive tumors in rats with a high brain-to-plasma ratio, and permeability independent of P-glycoprotein transport [678]. Moreover, despite heterogeneous intratumoral distribution, alectinib delayed tumor growth in an NB mouse model, leading to increased survival, providing an option for future clinical treatment [679,680,681]. An interesting point in this regard is that Alectinib may improve sensitivity to chemotherapeutic since it increases the intracellular accumulation of ABCB1/ABCG2 substrates such as doxorubicin (DOX) and rhodamine [682]. Moreover, it has also shown effectiveness in combination with the histone deacetylase inhibitor vorinostat in NB harboring the ALK R1275Q mutation and after intensive radiotherapy for the treatment of a rare intraosseous RMS with FUS-TFCP2 fusion, evidencing the potential of this drug to treat extremely aggressive tumors [683,684].

**AEE-788.** This drug is an orally bioavailable bispecific EGFR/HER2 inhibitor that exerts significant anti-tumoral activities and radio-sensitizes EGFR-overexpressing cells [685]. By targeting this receptor, the compound efficiently reduced clonogenicity, proliferation and survival of EPN cells and prolonged the survival of tumor-bearing mice, probably due to the increase in apoptosis of endothelial cells (as shown by others in cutaneous cancer xenografts) [686,687]. AEE788 also inhibited cell proliferation and prevented epidermal growth factor- and neuregulin-induced HER1, HER2 and HER3 activation in chemosensitive and chemoresistant (cisplatin selected) MB cells in vitro and in vivo [688].

**Crizotinib (Xalkori^®^).** This drug is an orally available aminopyridine-based ATP-competitive inhibitor of ALK that has shown positive results against NSCLC [689]. In turn, in pediatric tumors, growth-suppressive activities have been reported in some tumor types, such as PA, EPN, EWS and MB [160,690,691]. This drug was also able to induce apoptosis and autophagy in a dose-dependent manner in RMS cells, reducing cell migration and invasion, as well [692]. However, despite these promising results, this compound lacks clinical significance in patients with FOXO1-rearranged ARMS [693]. Similarly, crizotinib responses in NB are variable and mostly dependent on the mutation variants present in the tumor, considerably limiting its applicability [694,695,696]. Moreover, the literature widely illustrates that despite initial effectiveness, the vast majority of tumors treated with this compound will develop resistance within a few years [697].

**Capmatinib (Tabrecta^®^).** This compound is an orally bioavailable inhibitor of c-MET [597]. The information about the effects of this compound in pediatric oncology is limited. There are reports of its action in pediatric HGG in which this compound appeared to be more efficient than crizotinib in terms of specificity, potency and brain availability, resulting in a higher cellular response compared to crizotinib treatment in vitro and in vivo [698]. Nevertheless, in a phase I dose escalation study that included EWS and OS patients, only mild responses were observed [699].

**Tepotinib (Tepmetko^®^).** This compound is a phenylmethyl-pyrimidine derivative developed to disrupt MET phosphorylation that received approval from the FDA and the Japanese Ministry of Health, Labour and Welfare for the treatment of patients with metastatic NSCLC harboring METex14 skipping alterations who progressed following platinum-based cancer therapy [700]. According to PubChem (CID 25171648), this compound has been investigated in the treatment of neuroblastoma.

**PF-04217903.** This compound is an ATP-competitive small-molecule inhibitor with 1000-fold selectivity for c-MET compared with more than 150 kinases, making it one of the most selective c-MET inhibitors described to date. In vitro, it inhibited tumor cell proliferation, survival and migration/invasion in cell lines where c-MET is activated by different mechanisms, including c-MET gene amplification, HGF/c-MET autocrine loop formation and c-MET overexpression [701]. In vivo, oral administration or subcutaneous minipump infusions led to a robust tumor growth inhibition at doses of 30 mg/kg with suitable tolerability. Reductions in microvessel density were also observed [701]. Considering pediatric tumors, similar results were obtained when two highly metastatic OS cell lines were injected by tail vein into immunodeficient mice. In this experiment, mice were treated with PF-04217903 (30 mg/kg) or vehicle control by gavage 5 days on and 2 days off for 30 days. Mice injected with MNNG-HOS cells (which has constitutively activated MET) treated with PF- 04217903 had a tenfold reduction in the number of metastatic nodules, while those with injected MG63.2-derived tumors (which have high levels of total and phospho-MET) had a 37% reduction in nodules compared to control mice [702]. This compound has also shown potential for the treatment of malignant peripheral nerve sheath tumors in NF1 patients [703].

**Tivantinib.** This compound, also known as ARQ 197, was described as an orally bioavailable small-molecule c-MET inhibitor with antitumor activity. Tivantinib inhibited cell viability with similar potency in both c-MET-addicted and nonaddicted adult carcinoma cells, pointing to alternative mechanisms of action [704]. Despite this, the failure of a pioneer a phase I clinical trial in pediatric tumors was attributed to the lack of selection for MET amplification during patient enrollment. In the study, which comprised 36 patients, including 4 glioma, 4 MB, 4 EPN, 4 EWS, 4 OS, 3 RMS, 2 WT and 2 NB, suboptimal responses were achieved when tivantinib was given with food to children with refractory solid tumors is 240 mg/m^2^/dose. Moreover, while the drug was well tolerated, its pharmacokinetic profile was also variable, discouraging further investigation in this setting [705]. However, two of those patients (alveolar soft part sarcoma) who responded to tivantinib administration and were transitioned to a follow-up protocol experienced extended progression-free survival receiving 360 mg twice every day without adverse events [706].

**Lorlatinib (Lorbrena^®^).** This small molecule represents an orally available, ATP-competitive inhibitor developed by Johnson et al., and further investigated for the treatment of ALK-positive NSCLC [707]. Also named PF-06463922, the drug has shown minimal toxicity in adults and there has been much interest in its prospective use in NB treatment. In this regard, Infarnato et al. described higher potency of PF-06463922 across ALK variants in a panel of 10 NB cell lines, with IC_50_ values for inhibition of F1174L- and F1245C-mutated ALK significantly lower than those seen for its predecessor, crizotinib (0.2–10 nmol/L) [708]. Moreover, this compound at 10 mg/kg/day induced complete tumor regression in xenograft mouse models of NB, and in (PDX) harboring the crizotinib-resistant F1174L or F1245C mutations within 3 weeks [708]. Similar 10-fold lower IC_50_ values were obtained by Guan et al. (2016). In another group of cell lines, PF-06463922 inhibited growth, reduced levels of tyrosine 1278 (Y1278) phosphorylation on ALK, and induced apoptosis. Comparatively, treatment reduced tumor volume in subcutaneous and orthotopic xenograft models of NB, as well as in the Th-ALKF1174L/MYCN-driven transgenic NB mouse model [709]. PF-06463922 has also been tested sporadically in patients affected with NB. Two recent articles portray favorable responses in a 3-year-old boy with ALK-fusion-positive HGG and an adolescent with relapsed, refractory, metastatic ALK F1174L-mutated NB. The first, considering that the compound is able to cross the BBB, was treated through a nasogastric tube at a dose of 95 mg per square meter of body surface area once daily [710]. Histology after tumor resection showed a marked decrease in the proliferative index of the tumor and since the tumor was not seen on postsurgical MRI, therapy stopped. After 6 months, metastatic lesions were identified on cranial nerve VII and treatment was restarted at a dose of 95 mg per square meter administered by mouth once daily, achieving a near-complete response after 1 month [711]. In the second case, the patient had already shown no response to the first-generation ALK inhibitor crizotinib (240 mg/m2/dose given twice daily combined with the standard cytotoxic chemotherapy regimen). The tumor was reduced with continuous 95 mg/m2/dose lorlatinib and the only significant side effect observed was grade 2 hypercholesterolemia. However, differentially from the infant, she relapsed after 13 months of treatment and died from progressive disease 3 months later [712].

**Ceritinib (Zykadia™).** Formerly known as LDK378, it is an oral ALK inhibitor that also targets insulin-like growth factor receptor IGFR, insulin receptor and ROS1. This compound was approved by the FDA through an accelerated process to treat ALK-positive metastatic NSCLC [713]. Preclinical studies in the pediatric setting have indicated antiproliferative effects and improved inhibition (11-fold) compared to crizotinib [714]. However, in an exploratory study with a panel of NB cell lines, it was noted that inhibition occurs irrespective of ALK mutational status, and cell lines that carry other driver mutations (i.e., MYC amplification) are sensitive to treatment as well. The same authors further treated a child with ALK-I1171T high-risk NB that was not responding to conventional treatment due to an underlying congenital genetic condition, Fanconi anemia. Monotherapy with ceritinib was well tolerated and resulted in tumor shrinkage and complete clinical remission including all metastatic sites [715]. This compound can be given with food and penetrates the human brain, and thus presents itself as an option for the treatment of CNS tumors with ALK alterations such as EPN and MB [715,716,717,718]. However, in orthotopic PDX (from a 10-year-old boy with a multiple recurrent GBM), it was observed that even though ceritinib-treated mice lived longer, the drug had only a moderate effect [719]. Monotherapy was also inefficient in treating a 16-year-old patient with a long history of OS lung metastases, despite acceptable results in primary tumor cells of six other patients and the HOS cell line [720]. Similarly, Ceritinib treatment led to decreased cell proliferation, cell cycle arrest and apoptosis in a dose-dependent manner in a panel of RMS cell lines, all of which lack intrinsic ALK phosphorylation (PAX3-FOXO1-positive Rh30, Rh41 and -negative Rh18 and RD cell lines). The work showed that the compound affects the IGF1R signaling pathway without effects on the migratory ability of cells. Moreover, in subcutaneous Rh41 xenografts, a reduction in tumor growth was observed after approximately 2 weeks, albeit subsequent evaluation of tumor characteristics showed no difference in proliferation or vascularization between the treatment groups and controls [721]. Others also showed that even though LDK378 reduces cell viability and induces cell death in RMS cell lines at low micromolar concentrations irrespective of ALK expression levels or phosphorylation status, cells are far less sensitive compared with Karpas 299 non-Hodgkin’s lymphoma cells carrying the NPM–ALK fusion gene [722].

**Brigatinib (Alunbrig^®^).** Originally named AP26113, this next-generation ALK inhibitor was first described in 2016 and is considered highly CNS-penetrant [723,724]. This compound was granted approval for the treatment of patients with metastatic ALK+ NSCLC and intolerance to crizotinib [725]. In an NB setting, preliminary indication of efficacy was observed after exposure of several NB cell lines, including CLB-BAR (MYCN amplification, ALK (Δ4-11) and amplified, ALK addicted), CLB-GE (MYCN amplification, ALK (F1174V) amplification, ALK addicted), IMR32 (MYCN amplification, WT ALK) and CLB-PE (MYCN amplified, WT ALK), in which treatment inhibited cell growth and ALK phosphorylation in a dose-dependent manner. However, while IC_50_ values varied between 75 and 100 nM in ALK-addicted cell lines, the compound was unable to inhibit growth of both non-ALK addicted NB cell lines, IMR32 and CLB-PE. The effects of brigatinib were further validated in vivo through two complementary models. The first used transgenic *Drosophila melanogaster* flies expressing two gain-of-function variants, F1174L and R1275Q, which disrupt the eye morphology, giving a “rough phenotype”. The authors showed that larvae grown on food containing Brigatinib displayed a concentration-dependent improvement of the rough eye phenotype. Then, brigatinib was used as a single agent to treat BalbC/NUDE mice bearing ALK-addicted CLB-BAR xenografts. In this model, the compound also showed to be effective, with robust and potent anti-tumor activity [726].

**Entrectinib (Rozlytrek^®^).** This compound (also called RXDX-101, NMS-E628, NMS-01191372, Rozlytrek) is a selective, oral tyrosine pan-TRK, ALK and ROS1 inhibitor that has demonstrated preclinical efficacy in tumors with NTRK1/2/3, ALK and ROS1 alterations [727]. This inhibitor can pass through the BBB and has clinically proven to be effective against primary and metastatic brain diseases, with no adverse off-target activity [728].

Entrectinib also displays promising anti-tumor activity in NB, evinced by diminished Ki-67 and activation of caspase-3 in ALK wild-type, amplified or mutated cell lines [729]. In vivo growth inhibition and substantially reduced phosphorylation in TrkB-expressing NB xenografts were also observed after treatment as a single agent or in combination with irinotecan or temozolomide (TMZ), eliciting increased EFS when compared to controls [730]. Moreover, the ability of entrectinib to inhibit p-TrkB, p-PLCγ, p-Akt and p-Erk suggested that this compound may have improved efficacy compared to other targeted inhibitors previously evaluated in NB [172]. However, despite durable responses in pediatric patients with intracranial tumors or NB harboring NTRK1/2/3 or ROS1 fusions, its utility may be hampered by the appearance of acquired resistance in this tumor type [730,731].

**X-396.** This compound, also known as Ensartnib, is an aminopyridazine-based second-generation ALK/MET inhibitor that holds much clinical promise with increased potency as compared with crizotinib and other second-generation ALK inhibitors such as alectinib and ceritinib [732]. X-396 significantly reduced growth (by 40% at a 3 nM concentration) and ALK phosphorylation in SY5Y NB cells that harbor ALK-F1174L. Biochemical IC_50_ values for MET inhibition were 2-fold higher [733]. Ensartinib was significantly more effective than crizotinib at inhibiting the intracranial growth of the SH-SY5Y NB model harboring the F1174L mutation [732]. Furthermore, the activity of X-396 administered alone or in combination with liposomes carrying ALK-siRNAs (that are active irrespective of ALK gene mutational status) was later tested in a mouse model by Di Paolo et al. (2011). These authors corroborated previous in vitro data with a second NB cell line (LAN-5) and showed that in subcutaneous NB models, the compound acted in a dose-dependent manner, with adequate bioavailability, moderate half-life, high mean plasma and tumor concentrations. Moreover, against human NB orthotopic xenografts obtained by implanting of Luciferase stably transduced NB cells, SH-SY5Y-Luc and LAN-5-Luc, into the adrenal gland of nu/nu mice, significant dose-dependent anti-tumor activity was also observed, with even more reduced tumors and prolonged survival with the combination with the liposomal formulation [734].

**Erdafitinib (Balversa^TM^).** This compound is an oral pan-FGFR inhibitor with quinoxaline structure [735]. Known as JNJ-42756493, this compound is already approved by the FDA for the treatment of advanced or metastatic urothelial carcinoma, and is now under clinical trials that also include childhood CNS tumors [736]. It inhibits FGFR1/2/3/4 with increasing IC_50_ values of 1.2, 2.5, 3.0 and 5.7 nM, respectively [737]. This compound inhibited proliferation on five different NB cell lines (SK-N-AS, SK-N-BE(2)-C, SK-N-DZ, SK-N-FI and SK-N-SH) as monotherapy, but showed variable synergistic, additive and antagonistic effects when combined with commonly used cytotoxic agents such as cisplatin, vincristine and doxorubicin [738]. Additionally, IC_50_ for FGFR4 inhibition by this compound on the A-204 RMS cell line was determined as 4.5 nM, while treatment of mice xenografts resulted in a 58% volume reduction after 21 days of treatment with daily doses of 30 mg/kg [735].

Erdafitinib has also been tested alone and in combination with cisplatin, vincristine and radiotherapy on the SHH-MB cell lines DAOY and UW228-3. Under all conditions, the cell lines showed dose-dependent decreases in viability and proliferation after 48 and 72 h [739].

**Dovitinib.** Also known as TKI258, this is a multi-targeted tyrosine kinase inhibitor with potent activity against FGFR1/3, VEGFR1/2/3 and to different extents, PDGFR-β, Flt3, c-Kit and CSF-1R, that showed promising results as an antitumoral and antiangiogenic compound [658]. This compound is already in clinical trials in adult patients [740]. However, in pediatric neoplasms, information about preclinical studies is limited. Preliminary results of Dovitinib in NB cells, which express high levels of FGFR, indicated anticancer-activity in this tumor type [741]. Similar results were reported for RMS, albeit it was demonstrated that this inhibitor is not as potent as other FGFR inhibitors (i.e., ponatinib) [656,742]. In addition, due to its ability to cross the BBB, this compound has been indicated as a suitable candidate for the treatment of CNS tumors. In this regard, in vitro, it reduced the capacity of EPN cells to re-adhere and proliferate in a dose-dependent manner [658]. In DIPG and GBM, however, true effects on viability were observed at high dovitinib concentrations (>400 nM) [743]. Of note, others showed that despite killing glioma cells in vitro (up to 55% of cells at the assay end point), the drug exerted minimal anti-tumoral effects in vivo, suggesting a microenvironment-mediated therapeutic resistance mechanism [744].

**Masitinib (Masivet^®^).** This compound, also known as AB1010, is an orally administered, novel, potent and selective phenyl aminothiazole-type tyrosine kinase inhibitor of KIT, used in the treatment of canine mast cell tumors acting as a blocker of mast cell degranulation, cytokine production and migration of bone marrow cells [745,746]. Masitinib is under clinical investigation in several human malignancies that harbor similar canine KIT mutations (i.e., gastro-intestinal stromal tumors, ovarian and prostate cancer). In fact, this inhibitor acts on several mutated forms of KIT, and other receptors, including PDGFR, FGFR3 and focal adhesion kinase (FAK) [747,748]. Noteworthy, a brain tumor xenograft model using pediatric GBM cells suggested that masitinib may potentiate the effects of TMZ, providing decreased tumor growth relative to either drug used as a monotherapy [749].

### 4.2. PI3K/AKT/mTOR Pathway Inhibitors

**Everolimus (Afinitor^®^).** Everolimus (Afinitor, Novartis) is an orally administered rapamycin derivative approved by the FDA and the European Medicines Agency for the treatment of RCC [749]. This compound reduces tumor cell proliferation and induces apoptosis and autophagy through the phosphorylation inhibition of mTOR [750,751]. Preclinically, the combination of everolimus with sorafenib yielded enhanced antiproliferative and proapoptotic effects, potentiated antiangiogenesis and reduced the metastatic potential of OS [751]. Prolonged exposure to everolimus also improved the CNS retention of dasatinib and extended the survival of mice bearing pediatric high-grade glioma tumors [752]. Comparatively, everolimus is synergistic with carboplatin in low-grade glioma models [753]. However, in the literature, there is significantly more information about clinical experience because, since its approval, everolimus has become widely accepted by the medical community where treatment options may be limited. One major clinical example involves subependymal giant cell astrocytomas (SEGA), tumors that are frequently diagnosed in patients with tuberous sclerosis complex (TSC) [754]. Loss of function of either TSC1 or TSC2 leads to downstream constitutional activation of the mTOR complex [755]. Besides surgical excision, patients with large or recurring SEGAs did not have robust treatment options, and Everolimus has been shown to induce tumor shrinkage and presents additional clinical benefits including seizure control [756,757].

**Palomid-529.** Also known as RES-529, this compound is a small-molecule drug dual novel inhibitor of mTOR complex 1 (mTORC1) and mTOR complex 2 (mTORC2). Palomid 529 likewise inhibits both VEGF-driven and bFGF-driven endothelial cell proliferation [758]. Due to its potential to penetrate the BBB without restriction by the ABCB1 and ABCG2 efflux transporters, the anti-glioma effects of this drug have been investigated [759,760,761]. In childhood cancer, a single report proved a potent inhibition of viability, cell cycle progression and proliferation of the OS cell line U2OS [762].

**OSI-027.** This compound is an orally bioavailable selective ATP competitive inhibitor of mTOR and off-targeted PI3Kα (100-fold selectivity for mTOR relative to PI3Kα) that has been studied in the treatment of many tumors [763]. OSI-027 is active in vitro against cell lines and primary cells of pediatric pre-T-ALL, with superior efficacy to rapalogs and in vitro synergy with a number of conventional cytotoxic agents [181]. Preliminary studies combining OSI-027 treatment with alpelisib demonstrate similar antineoplastic results inhibiting PI3K/mTOR signaling MB, EWS and RMS cell lines [764,765,766,767].

**VS-5584.** This dual inhibitor of mTORC1/2 and class I PI3-kinases has shown antitumor potential in a broad spectrum of tumor types in vitro and in vivo [768,769,770]. Noteworthy, evidence supports that this compound has an active role in reducing stem cell viability in multiple mouse xenograft models of human cancer (30-fold more potent compared to non-stem cells) [769]. Thus, the activity of VS-5584 was recently explored in OS, in which treatment dramatically suppressed growth and cell migration and synergized with CCT128930, an AKT2 inhibitor [771,772,773]. In the same way, this drug is cytotoxic, showing apoptosis induction and a robust limitation of the colony-forming ability in NB cell lines. Delay of tumor growth was also observed in mice subcutaneously inoculated with BE(2)-M17 cells and treated with VS-5584 (25 mg/kg, three times per week) for 2 weeks [773].

**Dactolisib (BEZ235).** This drug, also called BEZ235 or NVP-BEZ235, is a reversible PI3K/mTOR inhibitor belonging to the imidazoquinoline class already tested in a variety of cancers in preclinical studies. In sarcomas (EWS, OS and RMS), dactolisib showed promising results in vitro, such as a reduction in cell proliferation, G1 cell cycle arrest and decreased in cell migration [774,775]. Interestingly, it was also shown that BEZ235 elicits strong cytostatic effects in EWS cells and results in a global modulation of the transcriptome affecting other pathways related to splicing and metabolism. This drug also reduced the expression of EWS/FLI1 by 50%, reinforcing its potential for EWS treatment [773]. However, its capacity to induce apoptosis is uncertain. Mild results were obtained by Giorgi et al. (2018), and when OS cells were treated with a similar inhibitor range, U2-OS and MG63 presented no significant differences in apoptosis induction, although the drug was efficacious with either doxorubicin or vincristine [774,775,776]. In RB, GBM, and MB, decreased viability and proliferation in a dose-dependent pattern was observed in most cell lines [777,778,779,780]. When tested in vivo, dactolisib could reduce tumor volume, vascularity and metastasis and improve animal survival, especially when combined with other drugs, such as topotecan, carboplatin, vincristine or the SMO inhibitor LDE225 [773,781,782,783].

**SF-1126.** This is a pan and dual first-in-class soluble PI3K/mTORC inhibitor that exhibits antitumor and antiangiogenic activity against several malignancies [784,785]. In the literature, there are few reports of this inhibitor in pediatric preclinical models. SF-1126 promoted a decrease in cell viability of a panel of EWS cell lines and CD15+ stem cell population in SHH-driven MB [786,787]. Moreover, SF1126 has been shown to enhance the cytotoxicity of doxorubicin in NB cells, leading to p53-mediated activation of apoptosis [788]. Treatment of NB tumors with SF1126 also reduced MYC expression and inhibited growth in vivo, leading to tumor shrinkage and reduced neovascularization [789].

**Triciribine.** Triciribine is a pan-AKT 1 inhibitor with anticancer effects in various tumor types [790]. This compound has been shown to decrease the survival of SH-SY5Y NB cells in both 2D and 3D culture models, affecting the migratory abilities of their sphere-forming units [791]. Triciribine demonstrated activity in EWS cell lines as well, with a mean IC50 of 24 μM, with robust synergy when combined with dasatinib; however, it did not affect tumor growth in vivo [792]. Moreover, Smeester and colleagues (2020) tested this drug in OS and showed that ATK inhibition in HOS and SJSA-1 cell lines leads to decreased cell proliferation, migration and colony capacity, and increased apoptosis. Further assessment in an orthotopic OS model also demonstrated reduced tumor growth (volume and weight) and metastasis after triciribine 40 mg/kg three times weekly [793].

**Sapanisertib.** Also called MLN0128, INK-128 or TAK-228, this is an ATP-competitive mTOR inhibitor already tested for safety in an adult cohort [794]. In studies including pediatric models, this inhibitor has shown promising results in vitro, reducing cell viability and colony formation and inducing apoptosis in sarcoma cells (EWS, OS and RMS) without affecting human osteoblast and osteocyte cells (normal bone cells); effects were improved by combination with MK2206, an AKT-specific inhibitor [795,796]. Similarly, the inhibition of mTOR (oral gavage for 21 days-3 mg/kg twice daily 3 x/week in EWS and RMS, or 2.5 mg/kg, daily) in OS resulted in tumor volume reduction, without observable side effects [795,796]. Comparable results were obtained for brain tumors (MB, NB and GB), with reduced cell invasion at low concentrations [797,798]. Interestingly, it was also observed that sapanisertib promotes metabolic alterations, such as disrupting glutathione synthesis and reducing glucose and lactate (a common feature of cancer cells) [797,798]. When tested in murine models, it reduced tumor weight and size, improving animal survival [797,798,799]. However, in combination with trametinib (1 mg/kg; 3 x/week; p.o. + MAPK inhibitor; 1.5 mg/kg; 5 x/week; p.o.), despite showing antitumor effects (reduction in angiogenesis and improvement in animal survival), some adverse effects were observed, including weight loss and skin redness [800]. Finally, in RB models, sapanisertib inhibited growth and increased apoptosis, whereas it inhibited cell migration and angiogenesis [801].

**LY-2090314.** This drug belongs to the ATP-competitive class of GSK-3 inhibitors with limited activity against additional kinases. Preclinical data suggested partial anticancer activity as a single agent against solid-tumor-derived cancer cell lines in vitro and in xenograft models, although it seemed to potentiate platinum-based chemotherapy [802]. Only a single report of its activity against pediatric tumors was found in the literature. Kunnimalaiyaan et al. (2018) tested LY2090314 in a panel of NB cell lines with different genetic backgrounds: SH-SY-5Y (non-amplified MYCN or single-copy, wild-type TP53, F1174L ALK mutation), NGP (1p alteration, MYCN-amplified, wild-type ALK, TP53 mutated, MDM2-amplified) and SK-N-AS (1p deletion, MYCN single-copy, H168R TP53 mutation, wild-type ALK), and found that this GSK-3 inhibitor at nanomolar range promoted growth inhibition in a time- and dose-dependent manner irrespective of the cell line markers. Reduced growth resulted mainly due to apoptosis induction, evinced by a 2-fold increase in the expression of cleaved PARP and capsase-3/7 activity. Downregulation of survivin and cyclin 1 was also observed [803].

**Tideglusib.** This compound represents another GSK-3 inhibitor, although it acts in a non-ATP competitive manner. Evidence of its antineoplastic effects with a pediatric scope includes in vitro experiments in OS and NB cell lines. In both models, treatment showed a significant reduction in cell proliferation in a dose-dependent manner, cell cycle arrest, and apoptosis induction, even though micromolar concentrations are required to achieve comparable results to LY2090314 [804,805,806]. Nevertheless, inhibition of GSK-3 by Tideglusib importantly compromises stem cell characteristics of both cell types. In the OS, treatment decreases stem cell markers, including OCT4, CD133 and SOX2, while in NB, it decreases neurosphere self-renewal. In mice models, tideglusib treatment (10 or 20 mg/kg in OS- and NB-derived tumors, respectively) promoted a reduction in tumor growth with few side effects [804,806]. Of note, PDX-derived cell cultures of both variants of RMS (embryonal and alveolar) treatment with tideglusib substantially reduced β–catenin phosphorylation at 60 nM; however, tumor-bearing mice treated with 200 mg/kg of tideglusib daily by oral gavage did not benefit in terms of survival or myodifferentiation [807].

**MK-2206.** This compound is an orally bioavailable allosteric and non-ATP-competitive AKT inhibitor tested in several tumors [808]. In OS, for instance, this drug was able to induce cytotoxic effects both in vitro and in vivo [223,796,809,810]. Similarly, in NB cells, MK-2206 diminished cell viability and increased apoptosis in cells with high expression of FOXO3a [810,811]. In vivo, the drug promoted inhibition of tumor growth and increased animal survival, effects that were even improved by combination with etoposide [812]. Of note, EWS and RMS cells were not sensitive or had less sensitivity to AKT inhibition [809].

**Ipatasertib.** Also known as GDC-0068, this compound is an ATP-competitive pan-AKT inhibitor developed by Array BioPharma/Genentech Inc. Having similar activity against Akt-1 and Akt-3, it is effective against several tumor types [813]. So far, Choo and colleagues are the only group that has tested this compound in childhood sarcomas. The drug induced a reduction in cell viability; however, RMS cells were more sensitive to PI3K/AKT pathway inhibition than OS cells [814].

### 4.3. MAPK Pathway Inhibitors

**Sorafenib (Nexavar^®^).** Sorafenib is an inhibitor of VEGFR2/3, PDGFR, KIT, FGFR-1, RAF and RET, approved by the U.S. FDA for the treatment of unresectable HCC and advanced RCC [815]. In preclinical models of MB, this compound reduced cell viability and increased apoptosis in established cell lines and primary tumor cultures. Moreover, it induced cytoskeletal alterations that ended in impaired cell migration [640]. In vivo, sorafenib (100 μL of 10 μmol/L administered three times a week for five weeks) was able to reduce the volume of subcutaneous tumors [816]. Similar results were obtained NB, in vitro and in vivo. Of note, in this model, sorafenib also impaired angiogenesis and G1 cell cycle arrest [817,818]. Conversely, despite reducing initial viability in EPN and PA, growth-factor-driven rescue was also seen, reducing the potential of using sorafenib for treatment of these tumors [691]. Indeed, in pediatric patients with PA, sorafenib induced progressive tumor growth acceleration as a result of ERK upregulation, which resulted in premature termination of the study [819]. In bone sarcomas, dubious results were also observed. In OS, sorafenib treatment blocked cell proliferation and was able to reduce tumor growth in murine models [820,821,822]. However, other studies showed that sorafenib was only able to reduce tumor growth when combined with everolimus, or palbociclib, probably due to the capacity of sorafenib to induce mTORC activation [821,823]. At the same time, in EWS and RMS, sorafenib only showed efficacy when combined with doxorubicin or with ceritinib, respectively [823,824].

**Regorafenib.** Also called BAY 73–4506, this is a new-generation multi-tyrosine kinase inhibitor that already showed antitumor and antiangiogenic effects. This inhibitor diminished cell proliferation in a cell line panel from the Innovative Therapies for Children with Cancer (ITCC), which includes 5 MB, 7 EWS, 7 NB, 7 OS and 7 RMS cell lines [610]. Regorafenib also induced cell cycle arrest and promoted apoptosis in NB cells [612]. In vivo, 10 mg/kg/d or 30 mg/kg/d treatment resulted in tumor growth inhibition in RMS, EWS and NB orthotopic models and improved EFS in EWS, NB, OS and RMS [610,825,826].

### 4.4. Cyclin-Dependent Kinases Inhibitors

**Milciclib.** This is a second-generation ATP competitive pan-CDK inhibitor, developed by Tiziana Life Sciences, that also acts on TRKs (from tropomyosin receptor kinase A) [827,828]. In preclinical trials performed in different tumors, such as MB and gliomas, it showed promise given its ability to cross the BBB, even though there are reports of MDR transporters limiting the penetration into the brain [829,830,831]. Moreover, MYCN-amplified the Grp3-MB cell lines MB002, Sd425 and D283 and the *MYCN*-amplified NB cell line Kelly are particularly sensitive to MILCICLIB treatment, evinced by cell cycle arrest and massive apoptosis [829].

**Terameprocol (CINelim™).** This is a semi-synthetic inhibitor developed by Erimos Pharmaceuticals LLC from a plant lignan, showing antiviral and anti-cancer potential [829,832]. The drug is considered a global inhibitor of the transcription process, which in turn acts by preventing, for example, the synthesis and activation of survivin, by competing with the transcription factor Sp1 for specific Sp1 DNA-binding domains within gene-promoter regions during DNA synthesis [833]. To date, only a single in vitro study (that included the childhood GBM cell line SF188) showed that this inhibitor was able to reduce the proliferation capacity of the cells in a dose-dependent manner, and showed synergism with TMZ under simultaneous exposure for 48 h. Increased effects were also observed when combined with ionizing radiation. Moreover, as expected, this compound induced significant arrest in the G0/G1 phase, decreasing the mitotic index and almost killing all cells at 30 μM [833].

**UCN.** UCN-01, or 7-Hydroxystaurosporine, is a synthetic derivative of staurosporine with antineoplastic activity that acts on AKT, CDKs and calcium-dependent protein kinase C (in an ATP-competitive manner), and is able to act synergistically with others [834]. In experimental models described in the literature, UCN-01 showed promising results by inducing apoptosis in leukemic and colon cancer cells; moreover, according to the pediatric tumors included in this work, this inhibitor also showed potential against OS tumor cells, reducing viability, proliferation and migration [835,836]. Similar results were observed in a panel of NB cell lines (with genetic backgrounds differing in MYC, p53 and BCL2 statuses), where this inhibitor was the most effective compound in reducing cell proliferation (compared to BiCNU, docetaxel, flavopiridol, staurosporine) and induced apoptosis measured through both caspase activation and caspase-3 and PARP cleavage [837].

**BMS-387032**. Also called SNS-032, this is a small aminothiazole molecule that acts as an ATP-competitive cyclin CDK inhibitor, especially for CDK2/7/9. Preclinical studies have shown that as a cell cycle blocker, it causes cytotoxicity and prevents tumor cell growth in several models [838,839,840]. Regarding pediatric tumors, this inhibitor showed positive results in the OS cell line U2-OS, evoking downregulation of RNA polymerase II Ser2 phosphorylation and some degree of caspase activation at all doses tested [839]. Similarly, this CDK inhibitor showed encouraging results in a panel of 109 NB cell lines, consisting of 19 parental cell lines and 90 sublines with acquired resistance to 14 different anticancer drugs. Doses between 58.3 and 14,615 nM were able to reduce viability in a great proportion of cell lines and impaired the growth of the multidrug-resistant cisplatin-adapted UKF-NB-3 subline UKF-NB-3(r)CDDP(1000) injected into the right flank of NMRI:nu/nu mice. Of note, p53 status did not affect the response of NB cells; however, ABCB1 expression conferred resistance to this drug [841,842,843]. Other interesting results, albeit not in child-derived cell lines, were also published, including the inhibition of hypoxia-mediated GBM cell invasion and cell-mediated capillary formation of HUVEC cells when co-cultured with U87MG cells in the presence of the drug [844,845]. Nevertheless, further studies with this inhibitor were stopped due to its high toxicity and low selectivity [846,847,848].

**Seliciclib (Roscovitine^®^).** Formerly known as Roscovitine, CYC202 or R-roscovitine, this is a selective ATP-competitive pan-CDK inhibitor that blocks cell proliferation in almost all phases of the cell cycle. Seliciclib is a potent inhibitor of CDK9/cyclin T, CDK7/cyclin H, CDK2/cyclin E and CDK1/cyclin B. The negative influence of seliciclib on CDK7 and CDK9 also portrays a role for this inhibitor in modulating RNA polymerase II CTD phosphorylation [848]. Its antitumor activity has been explored in a wide spectrum of hematological and solid malignancies as a single agent and in combination with other cytotoxic agents [848,849,850]. Among pediatric tumors, Roscovitine showed promising results, in EWS, where it was able to reduce cell proliferation and induce caspase-dependent activation (half minimal dose 10 μmol/L) in a panel of six cell lines, while it slowed A4573-derived tumor growth in mice after intraperitoneal injection [851]. Similar results were found in OS, with reduced proliferation and migration at doses up to 90 μM [437,851]. Moreover, in NB, the drug resulted concentration-dependent cytotoxicity, both in vitro and in vivo, with doses between 10 and 200 μM [851,852,853,854,855]. Roscovitine also reduced MB viability, with IC50 values of around 25 μM [856]. Moreover, treatment of Pzp53med cells (derived from a mouse Ptc+/−/p53−/− tumor) with 10 nM roscovitine resulted in reduced levels of E2F1, FASN, Bmi1, cyclin D2, cdk2 and cdk4. Synergistic effects were also observed when combined with C75, an inhibitor of FASN [857].

**Ribociclib (Kisqali^®^).** Also known as Kisqali^®^ (Novartis, Basel, Switzerland), this compound is a highly specific inhibitor of CDKs 4/6 that received FDA approval for use in the upfront treatment of hormone receptor-positive (HR^+^), HER2-negative breast cancer in 2017 [858]. With respect to the pediatric tumors, this compound demonstrated adequate results in EWS, causing cell cycle arrest mainly in combination with IGF1R inhibitors [859]. Moreover, in NB, this drug was able to reduce proliferation in vitro and in vivo, with doses between 0 and 10,000 nmol/L [331,860,861]. Most importantly, ribociclib showed high CNS penetration (>10 nM) in vivo, suggesting prospects for its use in the treatment of brain tumors. In this regard, oral doses of ribociclib inhibited RB phosphorylation, downregulated E2F target genes (CNE2, CCNA2, MKI67, TOP2A and PLK1) and decreased proliferation in group-3-MB mouse and human orthotopic PDX. Additionally, the combination of ribociclib and gemcitabine slowed tumor progression and metastatic spread and increased survival, warranting further investigation [862].

**Palbociclib (Ibrance^®^).** Also known as PD-0332991 (Ibrance^®^, Pfizer, New York, USA), this compound represents an ATP-competitor with selective potency against CDK4/6, approved by the FDA in 2015 [859]. The effects of this inhibitor have been assessed in several childhood tumors. In primary EPN cells, for example, it was able to reduce proliferation at 0.5 μM, with G1 arrest and reduced expression of CDC6, MCM2, MAD2L1, CDK2, BRCA2 and RAD51 [863]. Similar results were observed in NB, where this inhibitor reduced proliferation, inhibited colony formation in a dose-dependent manner and affected cell differentiation, tumor progression and metastasis in a preclinical chick embryo model [863,864,865,866]. Palbociclib has also been shown to be a new option for targeted therapy in childhood sarcomas. Perez et al. (2015), by treating a panel of 10 low-passaged sarcoma cell lines generated directly from patient samples and two commercial cell lines of heterogeneous origin and different molecular karyotypes (including liposarcoma, leiomyosarcoma, EWS, RMS and myxofibrosarcoma), determined IC_50_ values ranging from 8 to 26 μM depending on their levels of CDK4 expression. Moreover, palbociclib was active in vivo against subcutaneously engrafted CDK4-expressing sarcomas, although responses were negative in tumors displaying low levels of CDK4 and high levels of p16ink4a [867]. Strong decreased cell proliferation and G0/G1-phase arrest with decreased S/G2 fractions were also observed in leiomyosarcomas by another group [868]. Most interestingly, this compound is capable of inhibiting growth in sarcomas with different translocation backgrounds. For example, Palbociclib (100 mg/kg) was able to reduce the volume of tumors originated from an EWS sample with CDKN2A/B loss and FUS-ERG fusion implanted in the right chest wall of nude mice [869]. Additionally, satisfactory results were obtained after the treatment of a child with a refractory pediatric sarcoma harboring paracentric inversion on the short arm of chromosome X, resulting in the fusion of the BCOR and CCNB3 genes [870]. Regarding assays in OS, this inhibitor reduced proliferation and migration with doses of 0.04, 0.16, 0.625, 2.5 and 10 μM [393]. Migration and invasion have also been hampered by palbociclib in glioma cell lines, both in vitro and in vivo, with doses ranging between 10 nM and 10 µM [871,872,873]. Moreover, this inhibitor showed significant therapeutic benefit in mice after intracranial transplant of genetically relevant murine or human astrocytoma cells expressing BRAFV600E, and extended survival of animals when combined with PLX4720 (PubChem CID24180719) [874]. Similar results were also obtained in a DIPG with PDGF-B overexpression and Ink4a-ARF loss. Palbociclib induced cell cycle arrest in vitro and in vivo. However, in models engineered for PDGF-B expression with p53 deletion, the results were disappointing. Regarding survival, Palbociclib treatment prolonged animal survival by 12%, which was further increased by combinations with a previous single dose of 10 Gy radiation therapy [875].

**Abemaciclib (Verzenio^®^)**. This compound, under the name Verzenios^®^ (Eli Lilly, Indianapolis, USA), is a highly selective CDKs 4/6 inhibitor that acts by competing for the ATP binding site. This inhibitor is the most different from its peers (palbociclibe and ribociclibe), being more lipophilic and able to cross the BBB and penetrate breast tissue [859]. In addition, this inhibitor has potent activity against recurrent ER+/HER2- breast cancers [876,877] However, its clinical adverse effects are not well described [878,879,880]. Preclinical studies in pediatric tumors indicate effectiveness against EP, NB, EWS and OS [412,880,881,882]. Moreover, in gliomas, this inhibitor has been shown to be efficient in reducing cell migration and invasion, as well [883,884]. Finally, combining abemaciclib with other inhibitors, one of them being trametinib (MEK inhibitor), synergistically reduced the survival of the RAS-mutant RMS cell line RD. However, when PDX-bearing mice were treated with that combination, they exhibited progressive disease compared to the RMS standard-of-care regimen (irinotecan + vincristine) [885].

**AT-7519.** This is a potent pan-CDK inhibitor, acting on CDK1/2/4/6/9. Preclinical studies have shown a reduction in cell proliferation and induction of cell death in many cell lines, regardless of tumor origin [886,887,888]. In addition, this inhibitor showed promise for the treatment of MYC-amplified NB, evinced by apoptosis induction in vitro and dose-dependent growth inhibition in PDX, with improved survival and tumor regression in 86% of patients 7 days of treatment initiation [889].

### 4.5. Polo-Like and Aurora Kinases Inhibitors

**BI-2536.** This is an ATP-competitor dihydropteridinone that has proved to be more than 1000 times more specific for PLK1 than for other kinases [471,890]. This compound has been tested in several tumor cells, although reports for pediatric tumors are more uncommon. Our group showed that this compound reduces proliferation in up to 64% of cases, causes G2/M arrest and induces apoptosis after 24 h of treatment in the SF188 cell line, and it exerts the strongest radiosensitizing effect among all the cell lines tested [891,892]. Anti-mitotic and sensitizing to ionizing radiation effects were also demonstrated by us in MB cells, even though the results were comparable to other PLK1 inhibitors [893]. Others also showed that this compound suppresses self-renewal of patient-derived primary cells with high PLK1 but not low PLK1 expression, and it did not affect the growth of normal neural stem cells. Finally, BI2536 extended survival in MB-bearing mice [487,488]. Sensitizing effects were also observed for hyperthermia in the RB cell lines Y79 and WERI-Rb-1 [894].

With IC_50_ lower than 100 nM, BI 3526 was also able to induce cell cycle arrest at the G2/M phase and cell apoptosis in NB cells [492,895,896]. It has recently been proposed that this drug induces cell death by regulating the expression of the minichromosome maintenance complex components 2 and 10, which are involved in DNA replication and have been associated with poor outcome in other tumors [897].

Perturbation of normal mitotic progression by BI 2536 nanomolar concentrations (10, 50 and 100 nmol/L) also significantly decreased cell proliferation and clonogenic capacity, inducing mitotic arrest and aneuploidy in OS cell lines, resulting in caspase-independent mitotic catastrophe followed by necrosis [898]. Conversely, in another set of OS cell lines, apoptosis induction was validated through PARP cleavage and caspase activation. Irrespective of this, BI 2536-treated xenograft mouse models presented significantly smaller tumors compared with controls [899]. Moreover, in RMS, PLK1 inhibition by BI 2536 led to elevated ubiquitination and rapid proteasomal degradation of the PAX3-FOXO1 chimeric oncoprotein in vitro, whereas it reduced PAX3-FOXO1-mediated gene expression and elicited tumor regression in a xenograft mouse model [900]. Moreover, in this tumor type, this drug presented high antiproliferative activity when combined with Eribulin, a microtubule-interfering drug [901].

**NMS-1286937.** Also known as Onvansertib or NMS-P937, this novel PLK1-specific inhibitor has shown high potency at low nanomolar concentrations on a large number of cell lines, both from solid and hematologic tumors; in addition, differentially from other PLK1 inhibitors that compulsorily need intravenous administration, this small molecule can be administered orally [902]. Considering pediatric tumors, onvasertib has shown promising results in OS and MB. In the former, this drug proved to be highly active in both drug-sensitive and drug-resistant cell lines, except for cell lines overexpressing the multiple-drug-resistant transporter ABCB1 [903]. Results were also very promising in group-3 MB, which is characterized by PLK1 overexpression. In the study, treatment of D341, D425 and D458 cell lines resulted in reduced colony formation, cell proliferation, stem cell renewal and G2/M arrest. The half-maximal inhibitory concentrations varied from 4.9 to 6 nM. Other cell lines within the SHH subgroup needed 27.94 nM for comparable results. Moreover, onvansertib acted as a radiosensitizer, and showed marked time- and dose-dependent growth arrest of neurospheres and patient-derived short-term cultures. Most notably, onvansertib dramatically improved the median survival of orthotopic PDX models from 68 to 95 days [904].

**GSK-461364.** This compound is a second-generation and potent ATP-competitive thiophene amide PLK1 inhibitor. The anti-mitotic effects of this compound have been demonstrated in several tumors; however, it has been observed that its activity can be hampered by the overexpression of the multidrug resistance pump ABCB1 [905]. Preclinical findings in the pediatric setting include reduced viability after treatment in NB, MB and OS, in all of which it diminished growth and caused cell cycle arrest with massive apoptosis at a low-dose nanomolar range [906,907]. This compound also demonstrated a synergistic cytotoxic effect with paclitaxel, even though combination with methotrexate, cisplatin, vinblastine or doxorubicin was not that effective [907]. Conversely, this PLK1 inhibitor has been shown to be an effective radiosensitizer [908]. In vivo, GSK461364 treatment (50 mg/kg body weight intraperitoneally administered) strongly delayed the establishment of high-risk NB tumors in nude mice (by 22 days) irrespective of MYC status of the cell lines used, and significantly increased survival time in the treated group [906].

**Volasertib.** This drug, also known as BI 6727, is also a dihydropteridinone derivative that induces a distinct prometaphase arrest phenotype (polo-arrest) and subsequent apoptosis. Regarding pediatric tumors, in 2014, the NCI-supported PPTP Program published initial results about the use of BI 6727 in pediatric tumors. The systematic work presented in vitro results on 24 cell lines that included 4 RMS, 4 EWS, 1 GBM, 4 NB and several leukemias, concluding that the compound was effective without histotype selectivity. Then, the responsiveness of solid tumor xenografts (that also included OS and WT) using a dose of 30 mg/kg for 3 weeks was also tested. Volasertib was able to induce regression in only a minority of the models tested; however, significant differences in EFS distribution compared to control in 59% of the evaluable xenografts were observed, with better results for OS, WT and NB (the only one that showed objective responses) [909]. The results for OS were later validated in vitro by our research group, where treatment with BI 6727 not only led to growth arrest, triggered apoptosis and radiosensitized cells, but also it seemed to be more efficient in sensitizing OS cells to standard cytotoxics compared with GSK461364 [908]. Anti-mitotic effects were also observed in MB cells [893].

**Aurora kinase inhibitors.** Over the past two decades, several small-molecule inhibitors of Aurora kinases have been developed, most of which primarily target Aurora B. Despite not being approved or with clinical promise by the CanSAR platform, some of these inhibitors have shown promising results. MLN8237, for instance, was evaluated against a panel of EWS (*n* = 11) and NB (*n* = 17) cell lines with acceptable results in vivo [910]. The drug also inhibited growth uniformly in the majority of the cell lines from the PPTP in vitro panel, with IC_50_ values ranging from 49 nM to 61 nM. In vivo, EFS was 80% higher in treated animals compared to controls, showing even more auspicious results than those obtained for Volasertib [911]. More recently another AURK inhibitor, designated as PHA-680626, disrupted the AURKA/N-Myc, presenting a new alternative for the treatment of high-risk NB [912,913]. Additionally, AMG-900 (pan-aurora inhibitor) blocked MB cell proliferation and increased apoptosis and acted synergistically with the histone deacetylase inhibitor SaHa [914].

## 5. Kinase Inhibitors in Clinical Trials

**Figure 6:**
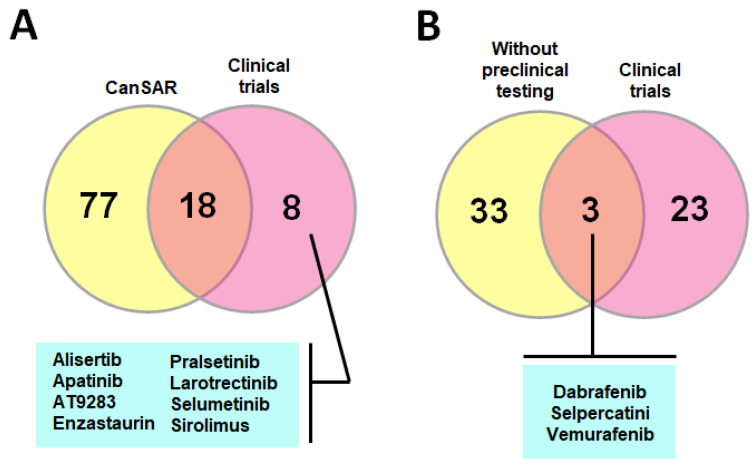
(**A**) Venn diagram showing the number of kinase inhibitors retrieved from the CanSAR platform versus those already tested in clinical trials. Around 30% of the compounds have been tested in patients. Of note, eight kinase inhibitors tested in clinical trials were not found in the CanSAR platform. (**B**) Venn diagram comparing kinase inhibitors found in the CanSAR platform without evidence of preclinical tests in pediatric cancer versus clinical trials. Three of the drugs are already tested in pediatric patients, without in vitro or in vivo evidence.

Clinical trials including children and using kinase inhibitors have been increasingly reported in recent decades. Among the 95 predicted compounds for our selected group of kinases retrieved through the CanSAR platform, for instance, 18 have already entered clinical trials (Figure 6A), most of which have focused on measuring cytotoxic effects on high-risk, refractory and recurrent tumors.

In order to gather an updated and comprehensive review on clinical data and outcome of pediatric tumors treated by these new TKIs compounds and already published to date, a PubMed search was performed (as per Oct 2022) using the following uniterms: ((cancer) AND (pediatric)) AND (kinase inhibitor). The following additional filters were set: clinical trial, meta-analysis, randomized controlled trial. In total, 233 articles were retrieved. Abstracts of the whole set of results were carefully read to exclude duplicated data/patients from the same clinical trial, articles where children and/or adolescents (<21 years of age) were not included or where clinical trials not testing kinase inhibitors were retrieved. A total of 53 articles met these inclusion/exclusion criteria, and were read and analyzed in full. Data on study design, study population, main clinical information and outcomes are summarized in Supplementary Table S5. The next part of this section does not review all studies on this subject, and also does not include clinical trials under development or in recruitment with unpublished data. Our main purpose was to gather and to discuss major sedimented data of clinical value and of clinical interest and applicability in this setting.

### 5.1. TRK—Tyrosine Receptor Kinases

#### 5.1.1. EGFR and VEGFR

Abnormal or disrupted angiogenesis is considered to be one of the hallmarks of cancer, and an increasing interest in targeting EGFR and VEGFR pathways has been observed in clinical oncology [915]. Unfortunately, most of these studies are focused on the adult population, and experience with these drugs in pediatric cancer is less robust. Yet, many of these compounds were tested in clinical trials that included children and/or adolescents with cancer, with variable results.

Regarding VEGFR inhibitors, at least three major molecule subtypes have been described. Type I VEGFR inhibitors exert their action as competing molecules to ATP [916]. Some examples of type I VEGFR inhibitors include pazopanib, axitinib, sunitinib, ponatinib and others. Type II inhibitors bind to the inactive “DFG-out” conformation adjacent to the ATP-binding site. Some examples of type II inhibitors include sorafenib and lenvatinib. Type III inhibitors lead to an irreversible binding of kinases at specific sites [917]. In addition, many of these VEGFR inhibitor molecules are under clinical evaluation in association with different inhibitors, particularly immunotherapy [918]. In addition, VEGFR2 inhibitors of dual action against other tumor-associated biomarkers are gaining much attention lately [919].

Inaba et al. (2011) evaluated the combination of sorafenib, a potent multikinase inhibitor, in association with cytarabine and clofarabine to treat relapsed or refractory childhood leukemia [916]. A total of 12 patients (<21 years of age; 11 with acute myeloid leukemia (AML) and one with early T-cell precursor leukemia) entered this phase I study. Of note, complete remission (CR) was obtained in 6 out of 12 patients, CR without complete blood recovery in 2 cases and partial remission (PR) in 1 case. Dermatologic, gastrointestinal (GI), metabolic and infectious adverse events were observed, and were more pronounced in the sorafenib higher-dose stratum. Grade 3 hand–foot skin reactions and/or rash were dose-limiting toxicities (DLTs). Sorafenib was also evaluated in a phase II trial, in association with everolimus to treat patients with progressive and unresectable high-grade osteosarcoma who failed standard treatment [920]. Although some encouraging initial results with sorafenib were observed earlier in this setting, a larger phase II study by Grignani et al. has shown some minor activity for selected cases, and the trial did not reach the 6-month progression-free survival target in at least 50% of patients [920]. Sorafenib was also evaluated in a phase I study that included refractory or relapsed hepatic tumors (hepatoblastoma or HCC) in children. The drug was used in association with irinotecan [921]. Six patients were evaluable for tumor response: two patients survived with no evidence of disease (NOD), one patient was alive with disease (AWD) and two patients DOD upon publication date. Radiation therapy and/or metastasectomies were offered after study protocol based on individual clinical needs. Increased grade 3 or 4 transaminase levels or neutropenia were reported.

Axitinib, a VEGFR1, 2 and 3 inhibitor, was also evaluated in a phase I study that included refractory solid tumors, as part of a Children’s Oncology Group (COG) trial and a pilot consortium trial ADVL1315. Nineteen patients were evaluated, with ages ranging from 9 to 17 years. Five patients achieved stable disease (SD), and a PR was observed in one case (an alveolar soft tissue sarcoma). The maximum tolerated dose (MTD) of axitinib was set at 2.4 mg/m^2^/dose [922]. Lenvatinib, a multiple oral tyrosine-kinase inhibitor against VEGFRs 1 to 3, RET, KIT, FGFRs and PDGFR-alpha were evaluated in a phase I/II pediatric and young adult trial for osteosarcomas [631]. The phase I study observed SD (some lasting for 23 weeks) as the best response obtained with Lenvatinib; the phase II study depicted two patients with partial response and thirteen children with SD. Although this single agent showed some activity in osteosarcoma, future studies will focus on the association of Lenvatinib with chemotherapy, or different molecules. Recently (September 2021), the FDA approved the use of cabozantinib for the treatment of patients (>12 years of age) with metastatic or locally advanced differentiated thyroid cancer (DTC), not amenable to receive iodine therapy, and who have failed different TKIs therapies. This approval was mainly achieved as a result of clinical findings of the COSMIC-311 study that observed prolonged progression-free survival in the group receiving the drug compared to the control (placebo) group.

Anti-EGFR therapy to treat pediatric malignancies has been less frequently evaluated in clinical trials. The Children’s Oncology Group (COG) evaluated gefitinib, an oral EGFR tyrosine kinase inhibitor, in children with refractory solid tumors [923]. Twenty-five patients were enrolled, and although the drug was well tolerated, only one patient showed partial tumor response in this study cohort. Gefitinib was also evaluated in concomitance to radiotherapy in newly diagnosed children with brainstem gliomas (DIPGs). Forty-three eligible patients entered this study. Although the vast majority of patients experienced rapid and fatal tumor progression, three patients remained free of tumor progression for more than 36 months, pointing to a possible benefit of this approach for a small subset of DIPGs [924].

#### 5.1.2. RET Inhibitors

Recently, RET-altered tumors were considered amenable to receive targeted therapy with RET inhibitors in a tissue-agnostic manner [925]. Vandetanib, a multi-TKI including RET inhibition, was evaluated in association with bortezomib in 22 patients (17 evaluable cases) with medullary thyroid cancer, with 27% showing partial responses [926]. Additionally, selpercatinib was evaluated in 42 patients with RET-fused tumors of different histologies other than lung and thyroid; durable antitumor activity across different tumor subtypes was observed, with only minor adverse effects [927]. Tissue-agnostic benefits of the use of RET inhibitors in patients with RET-fused tumors of different histologies were also confirmed with different drugs, such as Pralsetinib [928].

#### 5.1.3. ALK Inhibitors

A consortium phase I study coordinated by the COG evaluated the use of crizotinib for childhood cancer with refractory solid tumors or anaplastic large-cell lymphomas (ALCL). Seventy-nine children (aged 6 years or older) and adolescents were enrolled; tumor responses were more pronounced among patients with tumors with activating ALK aberrations [929]. In addition, Fukano et al. investigated the role of alectinib in primary refractory ALCL, or after relapsing, in a phase II study that included both children and adults. Eight out of ten enrolled patients responded to alectinib, with minor adverse effects described [930].

Moreover, Entrectinib, a potent CNS-penetrant inhibitor of TRKA/B/C, ROS1 and ALK, was also evaluated to treat children and young adults with solid or primary CNS tumors harboring NTRK, ROS1 or ALK aberrations [730]. In this phase I/II trial, the objective response rate (ORR) was 57.7% among 43 response-evaluable patients. Entrectinib shows a suitable safety profile and is effective as an option to treat pediatric patients with solid tumors harboring NTRK1/2/3 or ROS1 fusions.

### 5.2. PI3K/AKT/mTOR Pathway

The use of mTOR inhibitors, particularly everolimus, may be considered an important hallmark to treat children (>3 years old) with subependymal giant cell astrocytomas (SEGAs) associated with tuberous sclerosis complex (TSC) and not amenable to surgical treatment. This indication is largely derived from a phase III study (EXIST-1) that evaluated 117 patients in a double-blind placebo controlled trial, showing 50% tumor reduction exclusively in the treatment arm. The most frequent adverse events were oral ulcer and pyrexia; however, there was no treatment discontinuation due to adverse events [931].

### 5.3. MAPK Pathway

Abnormal, disrupted or constitutively activated MAPK pathways are involved in many pediatric cancers, particularly in low-grade gliomas (LGG). Recently, BRAF V600E-mutated tumors in children were eligible for agnostic treatment with BRAF plus MEK inhibitors [932]. Patients were randomized to receive either dabrafenib plus trametinib or standard chemotherapy. Among 110 treated children, complete and partial responses were reached in 47% of patients receiving the targeted therapy versus 11% for patients receiving chemotherapy alone. Of note, the clinical benefit rate, defined as complete, partial and stable disease lasting for more than 24 weeks, was 86% for trametinib plus dabrafenib versus 46% for standard chemotherapy [933]. Monotherapy with the BRAF inhibitor Dabrafenib was also previously evaluated in refractory or relapsed BRAF V600-mutated LGGs in children in a phase I/II study [934]. Among 32 enrolled patients (aged 1 to <18 years), the ORR was 44% and the 1-year PFS was 85%. In addition, adverse events (AE) were described in 91% of the participants; the most frequent AEs were fatigue, skin rash, dry skin and fever.

Disrupted MAPK pathways are also observed in patients with NF-1, where germline pathogenic neurofibromin mutations lead to the abrogation of the repressive function of this protein, with consequent activation of the PI3-K/AKT and RAS/MAPK cell signaling [935]. Besides the augmented frequency of LGG in patients with NF-1, plexiform neurofibromas are also frequently diagnosed in these patients, sometimes with life-threatening clinical consequences. Selumetinib, an MEK inhibitor, was evaluated in patients with symptomatic and inoperable plexiform neurofibromas [936]. Fifty children were enrolled in this phase II study that showed sustained tumor reduction, associated with clinical benefits. Improvements were observed in reducing pain and recovering motor function. However, 5 out of 50 patients discontinued treatment due to adverse effects possibly related to selumetinib, and 6 patients experienced disease progression while receiving the medication.

### 5.4. Cell Cycle Kinases

Ribociclib, an oral CDK4/6 inhibitor with pre-clinical evidence of action in different types of pediatric cancer, was tested either alone or in combination with chemotherapy in phase I and I/II studies, respectively [331,937]. Stable disease was the best response observed for both trials. The most common AEs were hematologic, including neutropenia, anemia and lymphopenia. More recently, palbociclib, a different oral CDK4/6 inhibitor, was evaluated in a phase I study directed at children and adolescents with progressive brain tumors. MTD of palbociclib was set at 75 mg/m^2^ (as monotherapy) for 21 days, followed by 7 days without medication. Neutropenia and thrombocytopenia were common AEs; no objective responses were observed among 35 enrolled patients [938].

In addition, different aurora-kinase inhibitors (AKIs) have undergone clinical trials in pediatric cancer. Thirty-seven patients were enrolled in a phase I COG study evaluating MLN8237, a selective AKI-A [939]. Myelosuppression, mucositis and hand–foot skin syndrome were common side effects. One PR and six prolonged SD were observed. AT9283, a different multitarget of AKIs A and B, was evaluated to treat pediatric patients with different types of solid tumors [940]. Of twenty-three evaluable patients, the authors described one confirmed PR and nine cases of disease stabilization after two courses of AT9283. More recently, Alisertib, a potent AKI-A, was evaluated in the pediatric population with both recurred/refractory solid tumors or leukemia (phase I). Five objective responses were reported, including two complete responses out of one-hundred and thirty-seven evaluable participants [941].

## 6. Final Remarks

Despite improvements, cancer is still responsible for 8% of all disease-related deaths in the pediatric setting [942]. Conventional chemotherapy not only is often ineffective but can also cause long-term complications that hamper the patient’s quality of life.

Over the past two decades, precision oncology and the advent of innovator small-molecule drugs or immunotherapy have revolutionized the treatment of many adult cancers, such as CML, GBM and certain types of breast carcinomas [12,943,944,945,946,947]. The tidal increase in genomic, epigenomic, transcriptomic, proteomic and biochemical data has enormously improved our understanding of the specific molecular signatures of pediatric solid tumors, as well as allowing the sub-classification of some tumor types, as is the case of MB and EPN [948], and the application of corresponding specific therapies. Indeed, we are currently in a transition state where the broadly applied decades-old and not always conclusively curative cytotoxic drugs are being gradually substituted by targeted ones, and in the near future, molecular technology will steer diagnosis and personalized treatment [949].

With over 500 kinases in the human genome regulating key biological processes, many members of this molecular family have gained scientific limelight in oncology and academic pharma. Herein, we provided an in-depth review of published data on the roles of the dysregulation of a selected group of kinases in tumor pathophysiology and corroborated their importance as therapeutic candidates in the context of pediatric solid tumors.

By 2020, the FDA had approved 52 small-molecule therapeutics that target nearly 20 different protein kinases (half of them are multikinase inhibitors and the majority target RTKs) [950]. Other drugs targeting an additional 15–20 protein kinases remain in clinical trials worldwide. Nevertheless, a total of 40 target kinases represents only 10% of the kinase superfamily, and most of those kinase-directed drugs have not been tested in pediatric patients. Still, critical challenges must be overcome in experimental oncology and the translation into clinical options. These challenges include the following:

(1) **Selectivity:** Most inhibitors developed so far target the ATP-binding site, meaning that they may act on multiple targets simultaneously and open new opportunities for the treatment of different tumor histologies [951,952]. Imatinib, for example, which has led to a significant increase in CML survival rates by selectively targeting the tumor-specific protein BCR/ABL, was included for the treatment of gastrointestinal stromal tumors (GIST), which are characterized by KIT-activating mutations [953]. Nevertheless, most inhibitors discovered to date have faced several adversities limiting their clinical use. First of all, the high sequence similarity in the ATP-binding sites frequently results in poor selectivity (refer to Figure 5 and Supplementary Figure S3) that may lead to undesired side effects. Moreover, these small molecules must compete with high intracellular ATP levels, leading to differences in potency when measured in vivo by biochemical versus cellular assays. In fact, many compounds inhibit their enzymes at nanomolar concentrations when measured biochemically, but only inhibit tumor cell growth under 3-fold higher concentrations [954]. Nevertheless, the increasing number of recognized kinase-specific structural features has allowed the emergence of superior non-ATP competitive kinase inhibitors that target other allosteric sites, which mostly act by inducing a conformational shift in the target enzyme, depleting its function [951,955,956,957].
(2) **Adverse effects:** Imatinib and dasatinib, for instance, are both licensed for the treatment of children with CML. Despite its undeniable benefits, and with the spectrum of side effects being comparable to what has been reported in adults (i.e., gastrointestinal toxicity, skin rash and muscle cramps), in a growing organism, imatinib treatment impairs longitudinal growth through the disturbance of osseous remodeling and inhibition of growth hormone secretion, which raises concerns about its lifelong use [958]. Moreover, despite the wealth of compounds that emerge on a daily basis, showing selectivity, potency and favorable pharmacological profiles, the probabilities for the translation into effective patient treatment for the great majority of them are extremely low. In this regard, less than 30% of the compounds approved or with clinical promise retrieved from the CanSAR platform have entered clinical trials in the pediatric setting (refer to Figure 6A). PLK1 inhibitors, for instance, despite the robust results obtained in vitro and in vivo, have demonstrated poor applicability due to severe hematological toxicity [959].
(3) **Mutational burden and lack of predictive biomarkers:** As stated before, the mutational identity may vary between adult and pediatric cancer, a feature that reflects in treatment response. Current treatments targeting ALK mutations in other cancers, for example, have not shown significant efficacy against NB. In this tumor, two hotspot mutations, at positions R1275Q and F1174L, occur in a high proportion of patients; tumors harboring the first are highly sensitive to crizotinib, while tumors bearing the second are resistant [960,961,962]. Moreover, as suggested by Bellantoni and Wagner (2021), childhood solid tumors may have fewer potentially targetable mutations, evinced by the inhibition of RTK for the treatment of OS, where it is necessary to target several relevant RTKs simultaneously to achieve desirable results [821]. Therefore, ground-breaking drugs for adult cancer may not be effective in the pediatric setting.
  In parallel, inhibitors are not effective if the target is not essential to drive tumor growth or does not represent a prognostic factor, as is the case of ROCK kinases. Even though these proteins have gained popularity and progressively been researched as targets for the development of novel anti-cancer drugs due to their association with metastasis and poorer patient survival in adult tumors, the influence of both isoforms on the prognosis of childhood cancer remains controversial [963].
(4) **Intrinsic and acquired resistance:** Resistance to targeted therapies is considered a largely inevitable hurdle that has a substantial impact on patients. Refractoriness to chemotherapy due to acquired F1174S ALK mutation in NB has been reported [964]. Likewise, the location of EGFR mutations significantly changes the effectiveness of EGFR; several mutations conferring resistance to EGFR tyrosine kinase inhibitors (such as T790M, L833V, A839T, V851I, A871T and G873E) have been reported [938]. Other examples include inadequate response to imatinib due to BCR-ABL1 kinase domain mutations that impart varying degrees of drug insensitivity, observed as underlying mechanism in 5–10% of adults and children with CML, bypassing pathway activation [965,966]. Moreover, despite an initial benefit of the targeted drug in molecularly well-defined tumors, patients inevitably experience tumor progression due to the development of resistance (i.e., Crizotinib in ALK-rearranged NSCLC population and CNS relapses) [967].
(5) **Lack of compounds designed specifically for childhood tumors:** In general, few pediatric patients with cancer are enrolled in clinical trials. The perception that adult studies can be generalized to children with similar diseases is a major obstacle. Consequently, most treatments are based on modifications of previously approved regimes for the adult population, and many compounds enter clinical trials without preclinical testing in pediatric oncology (refer to Figure 6B), which is mandatory to obtain a more accurate interpretation of its possible therapeutic potential in a certain cancer entity [968]. Moreover, pediatric cancer is rare, and even among patients with the same cancer type, there is often broad heterogeneity in terms of prognosis, molecular features or pathology. Therefore, few institutions have sufficient patients and the chances of every potential agent or combination being tested are reduced. Even so, priorities for funding are typically assessed according to the “burden of illness” for diseases, which is traditionally determined by disease frequency and mortality rate, leading to reluctance to distribute limited research funding to pediatric trials [969,970,971].

Regardless of the above challenges, kinase-based drug discovery has attained dramatic growth in the past 20 years. Although kinase inhibition represents a young therapeutic strategy compared with other traditional tactics, the FDA has approved a median of almost two small-molecule kinase inhibitors per year [952]. Thus, increasing numbers of targeted therapies are being tested for pediatric cancers, and many have shown undeniable success. Besides, the inhibition of kinases in normal cells can be clinically tolerated, presenting a therapeutic window that allows the softening of the acute side effects that generally lead to refusal and abandonment of treatment [972].

Moreover, as research advances, it has become clear that kinase inhibitors do not have to be absolutely selective. Crizotinib, for instance, was initially developed as an MET inhibitor, but later it was found to be even more efficient in cancers with ALK rearrangements. Additionally, molecularly targeted therapies are proving to be more effective in combination regimes to completely shut down the dysregulated pathway. As an example, it has been shown that everolimus improves CNS retention of vandetanib, dasatinib and sorafenib, which may have a great impact on the treatment of CNS tumors or brain metastases [751,752]. In the same vein, third- or fourth-generation inhibitors are being developed to avoid resistance and improve other biopharmaceutical properties such as brain penetration. These inhibitors, coupled with the increased ability to characterize tumors on molecular and genomic levels, will not only enable treatment refinement by identifying which patients may benefit most, but in the near future may conquer many diseases that are currently incurable.

## Data Availability

Not applicable.

## Supplementary Materials

The following supporting information can be downloaded at: https://www.mdpi.com/article/10.3390/pharmaceutics15020664/s1, Figure S1: Flowchart depicting the identification of databases used in the present study, describing the number originally identified, included and excluded, and the reasons for exclusions; Figure S2: Flowchart representing kinases of interest analysis in cell line dependency score and the identification of inhibitors, preclinical studies and clinical test describing the number originally identified, included and excluded, and the reasons for exclusions; Figure S3: Schematic illustrations of kinases druggability identified by the CanSAR database in the other kinase families, including the total number of compounds with predicted interaction capacity with each kinase, as well as FDA-approved drugs, and clinical candidates. Interaction networks of kinase inhibitors and associated binding proteins according to STITCH (‘search tool for interactions of chemicals’). Compounds are represented as pill-shaped nodes, while proteins are shown as spheres. Small nodes represent proteins of unknown 3D structures, while large nodes show proteins with known or predicted structures. Nodes that are associated to each other are linked by an edge: thicker lines represent stronger binding affinities. Networks were constructed considering a minimum required interaction score of 0.700, and based on associations reported in Curated Databases (gray lines), or on both Databases and Experimental/Biochemical Data (green lines). Purple lines represent functional links between proteins; Table S1: Differential expression of kinases (pediatric tumors vs normal tissue); Table S2: Knase expression and clinical features; Table S3: DepMap scores; Table S4: preclinical data; Table S5: Clinical trials.
