# Supplementary material for: Drugging Hijacked Kinase Pathways in Pediatric Oncology: Opportunities and Current Scenario
Source: Pharmaceutics. 2023 Feb 16;15(2):664. doi: 10.3390/pharmaceutics15020664 (PMC9966033; doi:10.3390/pharmaceutics15020664)

Supplementary Figures

DRUGGING HIJACKED KINASE PATHWAYS IN PEDIATRIC ONCOLOGY: OPPORTUNITIES AND CURRENT SCENARIO

Marina Ferreira Candido MSc[1], Mariana Medeiros MSc[2], Luciana Chain Veronez PhD[3], David Bastos BSc[4], Karla Laissa Oliveira BSc[4]; Julia Alejandra Pezuk PhD[5], Elvis Terci Valera PhD[3] and María Sol Brassesco PhD[5*]

[1]Department of Genetics, Ribeirão Preto Medical School, University of São Paulo, Ribeirão Preto, São Paulo, Brazil.

[2]Regional Blood Center, University of São Paulo, São Paulo, Brazil.

[3]Department of Pediatrics, Ribeirão Preto Medical School, University of São Paulo, Ribeirão Preto, São Paulo, Brazil.

[4]Department of Biology, Faculty of Philosophy, Sciences and Letters at Ribeirão Preto, University of São Paulo, Brazil.

[5]Anhanguera University of São Paulo, UNIAN/SP, Brazil.

*Corresponding author: María Sol Brassesco. Departamento de Biologia, FFCLRP-USP. Av. Bandeirantes, 3900. Bairro Monte Alegre. CEP 14040-901. Ribeirão Preto – SP. Brazil. Phone: +55 16 33159144, fax: +55 16 33154886, e-mail: solbrassesco@usp.br, orcid.org/0000-0003-4447-784X

Supplementary Figure S1: Flowchart depicting the identification of databases used in the present study, describing the number originally identified, included and excluded, and the reasons for exclusions.

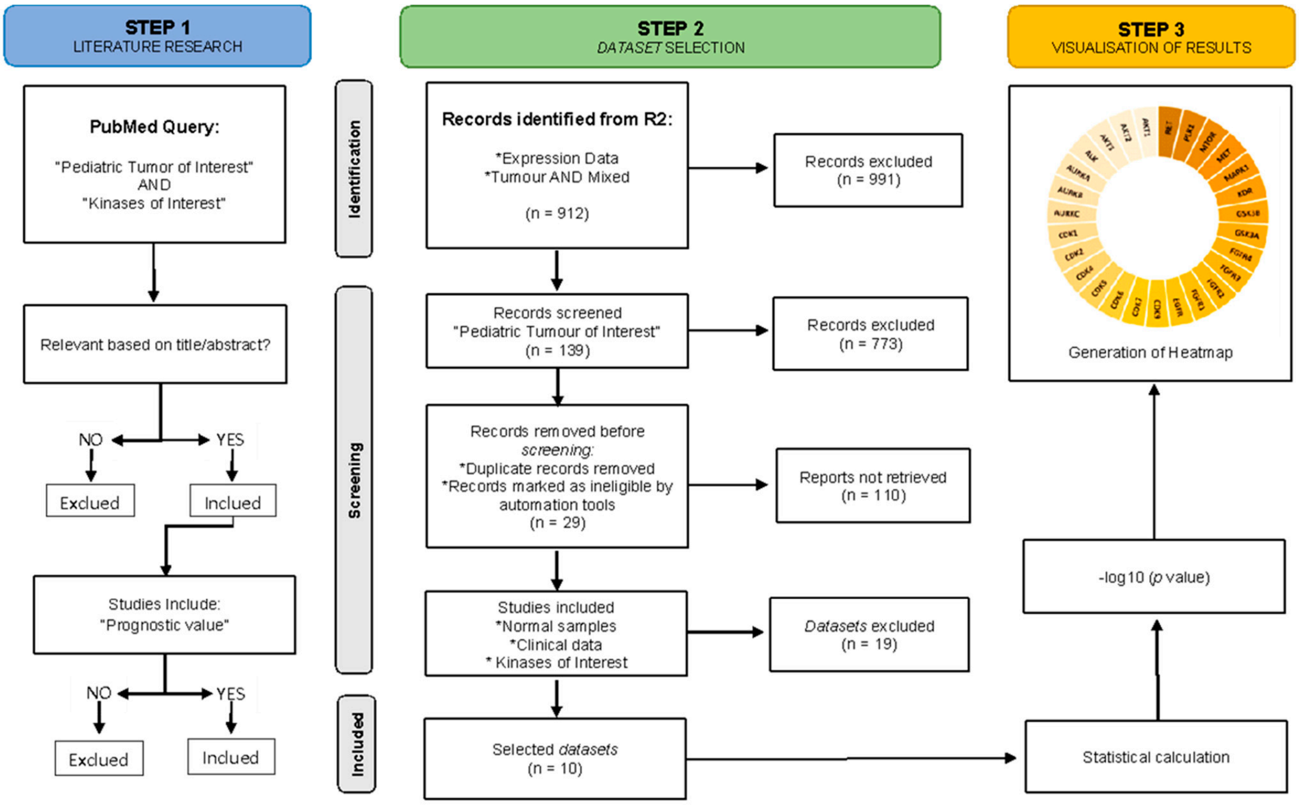

Adapted from: Page MJ, McKenzie JE, Bossuyt PM, Boutron I, Hoffmann TC, Mulrow CD, et al. The PRISMA 2020 statement: an updated guideline for reporting systematic reviews. BMJ 2021;372:n71. doi: 10.1136/bmj.n71. For more information, visit: http://www.prisma-statement.org/

Supplementary Figure S2: Flowchart representing kinases of interest analysis in cell line dependency score and the identification of inhibitors, preclinical studies and clinical test describing the number originally identified, included and excluded, and the reasons for exclusions.

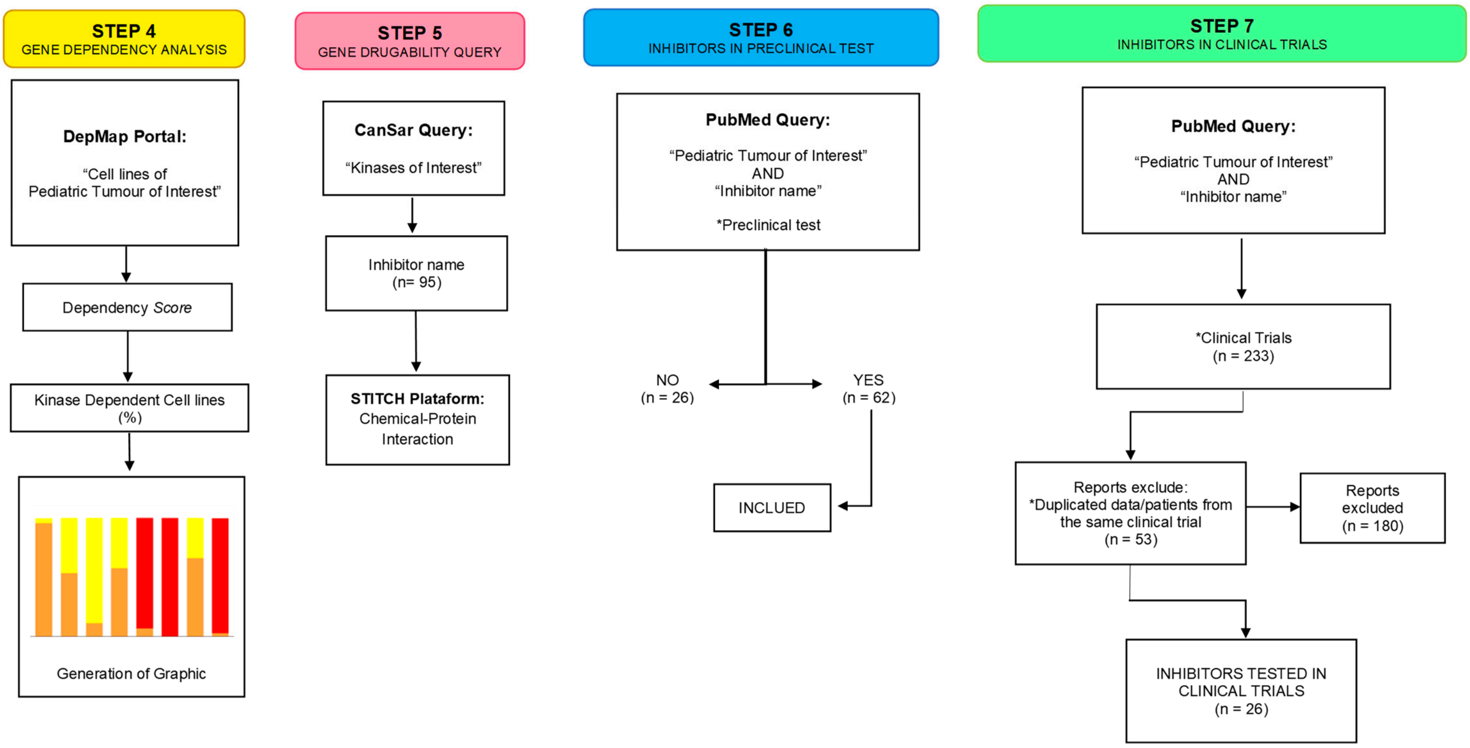

Adapted from: Page MJ, McKenzie JE, Bossuyt PM, Boutron I, Hoffmann TC, Mulrow CD, et al. The PRISMA 2020 statement: an updated guideline for reporting systematic reviews. BMJ 2021;372:n71. doi: 10.1136/bmj.n71. For more information, visit: http://www.prisma-statement.org/

Supplementary Figure S3: Schematic illustrations of kinases druggability identified by the CanSAR database in the other kinase families, including the total number of compounds with predicted interaction capacity with each kinase, as well as FDA-approved drugs, and clinical candidates. Interaction networks of kinase inhibitors and associated binding proteins according to STITCH ('search tool for interactions of chemicals'). Compounds are represented as pill-shaped nodes, while proteins are shown as spheres. Small nodes represent proteins of unknown 3D structures, while large nodes show proteins with known or predicted structures. Nodes that are associated to each other are linked by an edge: thicker lines represent stronger binding affinities. Networks were constructed considering a minimum required interaction score of 0.700, and based on associations reported in Curated Databases (gray lines), or on both Databases and Experimental/Biochemical Data (green lines). Purple lines represent functional links between proteins.

PI3K/AKT/mTOR inhibitors

	Compounds	Clinical promise	FDA approved
mTOR	6188	15	2
GSK3A	3291	0	0
GSK3B	7610	2	0
AKT1	6134	4	0
AKT2	2756	0	0
AKT3	1622	0	0

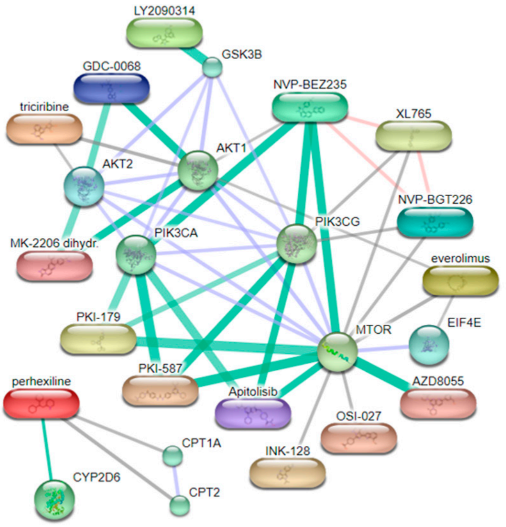

MAPK inhibitors

	Compounds	Clinical promise	FDA approved
MAPK1	7872	0	0
MAPK3	2718	0	0
RAF1	4764	3	4
PLK1	3242	5	0

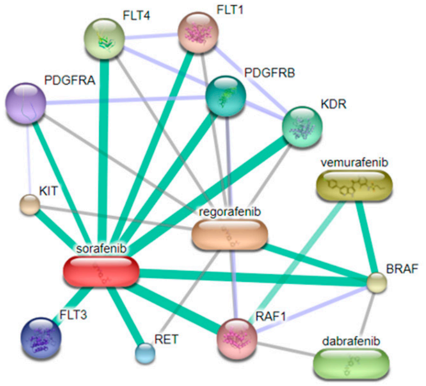

Cell cycle inhibitors

	Compounds	Clinical promise	FDA approved
PLK1	3242	5	0
AURKA	5927	0	0
AURKB	4558	0	0
AURKC	1167	0	0
CDK1	4509	8	0
CDK2	9410	12	0
CDK4	4244	7	4
CDK5	3829	4	0
CDK6	4244	1	4
CDK7	1929	4	0
CDK9	4973	7	0

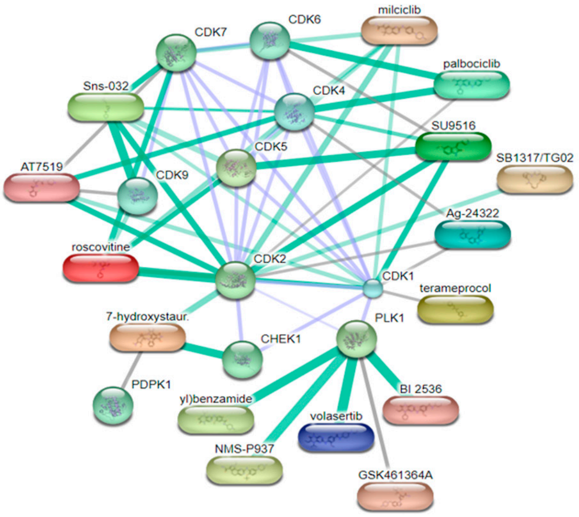

Supplement: Supplementary file 1 [file pharmaceutics-15-00664-s001.zip › Supplementary Figures.pdf]
